# Persistent inequities in neonatal encephalopathy: a 30-year global burden analysis (1990–2021)

**DOI:** 10.3389/fped.2025.1694958

**Published:** 2026-01-15

**Authors:** Yiming Yuan, Mingyue Zhao, Jincao Zhi, Zinan Guo, Jianyang Dong, Xiaoying Tian, Lin Feng, Yan Wang

**Affiliations:** 1 Hunan University of Medicine, Huaihua, Hunan, China; 2 Heilongjiang University of Chinese Medicine, Harbin, Heilongjiang, China; 3 Second Affiliated Hospital of Heilongjiang University of Chinese Medicine, Harbin, Heilongjiang, China; 4 Shenzhen University General Hospital, Shenzhen, Guangdong, China

**Keywords:** birth asphyxia, birth trauma, disability-adjusted life years (DALYs), global disease burden, neonatal encephalopathy, sociodemographic index (SDI)

## Abstract

**Background:**

Neonatal encephalopathy (NE) remains a significant cause of mortality and long-term disability in children under five, with pronounced global disparities in incidence and outcomes despite available interventions. This study aims to identify inequalities in the NE burden, with the goal of informing strategies to promote health equity and well-being in children.

**Methods:**

Using data from the GBD 2021 study, we analyzed NE incidence, mortality, and disability-adjusted life years (DALYs) across 204 countries from 1990 to 2021. Data were assessed by age, sex, region, and Sociodemographic Index (SDI). Trends were quantified using estimated annual percentage change (EAPC)**.**

**Result:**

Globally, NE incidence declined by 18.1%, mortality by 31.6%, and DALYs by 27.7% from 1990 to 2021. Males had a higher burden. A substantial disparity was observed: the age-standardized mortality rate in low-SDI regions was 22 times that of high-SDI regions. Western Sub-Saharan Africa and South Asia carried the highest burden. Low birth weight accounted for 58.4% DALYs of NE globally, with a disproportionate effect in low- and middle-SDI regions.

**Conclusion:**

Despite an overall reduction in the global NE burden, it remains disproportionately concentrated in resource-limited settings. Persistent disparities in low- and middle-SDI regions necessitate urgent, targeted interventions—including scaled-up perinatal care, strengthened health infrastructure, and region-specific strategies—to mitigate inequities. Middle- to high-SDI countries must address evolving epidemiological patterns. Equitable expansion of rehabilitation resources is critical to improving long-term outcomes and reducing disease burden.

## Introduction

Neonatal encephalopathy, a clinical syndrome in term and late preterm infants, is characterized by a significantly elevated risk of perinatal mortality and long-term neurological impairments, including cerebral palsy, movement disorders, an altered state of consciousness and seizure disorders (1). NE resulting from birth asphyxia and trauma is a major contributor to brain injury in term neonates (2, 3). Asphyxia is primarily induced by perinatal hypoxia, whereas traumatic brain injuries—often resulting from delivery-related procedures—can lead to intracranial hemorrhage or mechanical insult, culminating in significant developmental impairments. Asphyxia-induced NE constitutes the majority of cases, whereas traumatic NE is less frequent but associated with substantially higher rates of mortality and long-term disability in severe cases (4, 5). The global burden of NE is substantial, with an estimated incidence ranging from 3 to 8.5 cases per 1,000 live births, affecting more than 1.2 million newborns each year (6). Survivors are often faced with permanent neurological disabilities (7), which can range from mild to profound and persist from childhood throughout life (8). The incidence of NE in low-income countries is approximately 8–16 times higher than in high-income countries (7). Importantly, the number needed to treat is approximately 7–10 to achieve a single additional favorable neurological outcome. Neonatal hypoxic-ischemic encephalopathy (HIE) is typically an unforeseen acute condition that can present at both community hospitals and specialized referral centers with neonatal intensive care units (NICUs). Consequently, families have little time to prepare for such a diagnosis (8). Furthermore, various prenatal and societal factors, including socioeconomic status, maternal nutrition, and healthcare infrastructure, significantly increase the risk of mortality. Consequently, survival rates vary dramatically across different settings, and affected children, their families, and societies are compelled to contend with the profound burden of long-term neurodevelopmental impairment (9).

The Global Burden of Disease (GBD) study provides a comprehensive framework for quantifying disease burden, incorporating neonatal encephalopathy (NE) caused by asphyxia and trauma into a unified category. On one hand, this aggregation addresses the issue of global data heterogeneity arising from disparities in data collection capacities across regions, and the standardized methodology enables cross-regional comparisons to observe the epidemiological burden of the disease. On the other hand, these two subtypes share analogous clinical outcomes—characterized by high neonatal mortality and long-term neurodevelopmental disabilities—which synergistically exacerbate the overall disease burden. Ultimately, this unified classification aims to inform resource allocation and policy formulation, thereby mitigating the global burden of NE (10, 11). We analyzed incidence, mortality, and prevalence data from the GBD database from 1990 to 2021 to delineate the global epidemiological profile of NE. The health loss attributable to NE was assessed using Disability-Adjusted Life Years (DALYs), a composite metric calculated as the sum of Years Lived with Disability (YLD) and Years of Life Lost (YLL) (12). Furthermore, we integrated data on NE risk factors to provide a comprehensive overview of the trends in the global burden of NE. This analysis aims to provide policymakers and healthcare practitioners with critical insights into the burden of NE, to help mitigate persistent inter-regional inequalities, and to inform strategies for addressing the healthcare needs of individuals with NE and its long-term sequelae. The ultimate objective is to shift the focus from merely ensuring survival of children with NE to promoting their intact survival and long-term well-being, in alignment with the Sustainable Development Goals' target of reducing global neonatal and under-five mortality (13).

## Methods

### Study population

The study included patients diagnosed with neonatal encephalopathy (NE) attributed to birth asphyxia or trauma. The analysis was conducted across both sex and all age groups. The GBD database provides both both sex, complete and standardized all ages group categorizations, enabling comprehensive comparisons and analyses.

### Data collection

NE due to birth asphyxia and trauma was identified based on the ICD-10 codes: P01.7, P02–P03.9, P10–P15.9, P20–P21.9, P24–P24.9, and P90–P91.9. Conditions not falling within the specified coding range above, such as congenital malformations, chromosomal abnormalities, intrauterine infections, metabolic diseases, encephalopathy from non-traumatic causes, and neurological sequelae diagnosed after the neonatal period, are excluded from this study's analysis. Data were retrieved from the Global Health Data Exchange (GHDx) platform (http://ghdx.healthdata.org/). The search parameters included: “encephalopathy due to birth asphyxia and trauma” for cause; “incidence, prevalence, deaths”, “DALYs” for indicators; “all locations” for geographic coverage; “1990–2021” for years; “number and rate”for metrics; “male, female, and both” for sex; “age-standardized”, “all ages”, and “5-year intervals” for age; and “high, high-middle, middle, low-middle, and low SDI” for SDI categories. The study was conducted in accordance with standardized guidelines for cross-sectional analyses.

### Statistical analysis

Data from the GBD database were used to describe trends in incidence, prevalence, mortality, DALY rates, and associated risk factors of NE due to birth asphyxia and trauma across global, regional, and national levels between 1990 and 2021 (14). Metrics included numbers and age-standardized rates (ASRs), standardized to the GBD 2019 global reference population. No additional age-weighting or *post hoc* comorbidity corrections were applied, as these are inherently addressed within GBD modeling frameworks. Age-standardized incidence rates (ASIR), mortality rates (ASMR), and DALY rates (ASDR) were calculated and visualized using global heat maps. Temporal trends in ASRs were assessed using estimated annual percentage change (EAPC), calculated via log-linear regression: ln (ASR) = *α* + *β*(year). EAPC was derived as 100 × (e^*β* − 1), with 95% confidence intervals. Analyses were conducted in R (version 4.3.2) using tidyverse and base R functions. EAPCs for ASDR and ASPR were computed at global, regional, and national levels and visualized using choropleth maps (15). Patients were categorized into 10 age groups (<5, 5–9, 10–14, 15–19, 20–24, 25–39, 40–44, and ≥45 years) to evaluate temporal changes in age distribution from 1990 to 2021. The age-specific composition of NE-related deaths was compared globally between 1990 and 2021. All statistical analyses were conducted using R software (version 4.3.2; R Core Team) (16).

## Results

### 1. Global temporal pattern of neonatal encephalopathy

**Figure 1:**
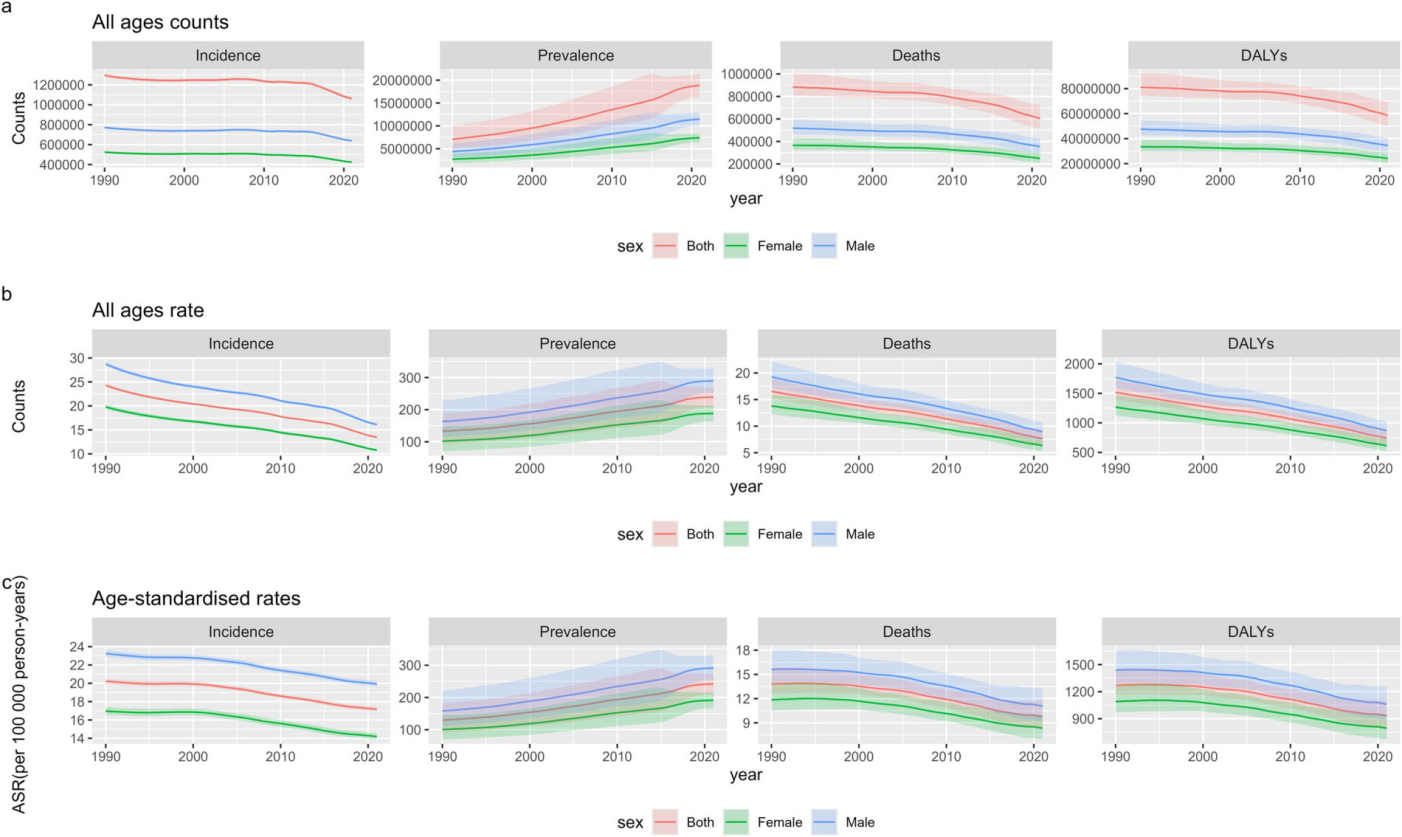
Global temporal pattern of neonatal encephalopathy due to birth asphyxia and trauma burden, 1990-2021 **(a)** counts of NE. **(b)** All ages rates of NE. **(c)** Age-standardised rates of NE.

Between 1990 and 2021, the global incidence of neonatal encephalopathy (NE) attributable to birth asphyxia and trauma exhibited notable fluctuations in both sex. The incidence declined from 1990 to 1998, rose during 1999–2007, and declined again from 2008 to 2021, with a transient peak in 2012. Overall, the trend was downward. The number of new NE cases decreased from 1,295,891 (95% UI: 1,276,553–1,314,239) in 1990 to 1,061,448 (1,047,815–1,076,766) in 2021 (Figure 1a; Supplementary Appendix 1). NE-related deaths (both sexes) varied moderately from 1990 to 2019. Mortality declined from 1990 to 2003, rose modestly between 2003 and 2005, and then continued declining through 2021. The number of deaths declined from 883,082 (95% UI: 809,080–1,004,097) in 1990 to 603,606 (511,191–725,271) in 2021. Total DALYs fell from 81,020,506 (95% UI: 74,272,388–92,120,587) in 1990 to 58,575,091 (50,032,891–68,835,375) in 2021. From 1990 to 2021, the age-standardized incidence rate (ASIR) of NE due to asphyxia and trauma declined consistently, from 20.2 (95% UI: 19.9–20.5) to 17.2 (16.9–17.4) per 100,000 (Figure 1c; Supplementary Appendix 3). The age-standardized mortality rate (ASMR) fell from 13.8 (12.7–15.7) to 9.8 (8.3–11.7) per 100,000, and the age-standardized DALY rate (ASDR) decreased from 1,271 (1,165–1,444) to 932 (796–1,102) per 100,000.

From 1990 to 2021, male infants consistently exhibited higher incidence, mortality, and DALY burdens of NE caused by asphyxia and trauma compared to females (Figure 1b; Supplementary Appendix 2). In 2019, male cases were 637,777 (95% UI: 629,259–647,596), 1.5 times that of females [423,674 (414,566–433,312)]. Male deaths reached 354,306 (293,054–422,742), 1.4 times higher than females [249,299 (207,718–297,289)]. Similarly, male DALYs were 34,414,411 (28,910,399–40,569,208), also 1.4 times that of females [24,160,972 (20,445,157–28,526,986)] (Supplementary Appendix 1). Between 1990 and 2021, ASIR, ASMR, and ASDR declined in both sexes, mirroring overall trends, yet remained consistently higher in males. In 2021, male ASIR was 19.9 (19.7–20.2) per 100,000, 1.4 times that of females [14.2 (13.9–14.5)]. Male ASMR was 11.1 (9.2–13.2), 1.3 times higher than females [8.3 (6.9–9.9)]. Male ASDR reached 1,060.2 (889.7–1,252.6) per 100,000, also 1.3 times that of females [795.4 (670.5–942.3)] (Figure 1c; Supplementary Appendix 3).

### 2. Region-level in age-standardized DALYs (ASDR), mortality (ASMR) of neonatal encephalopathy

Across all SDI-based regions, the burden of NE due to asphyxia and trauma has declined from 1990 to 2021. High-middle SDI countries experienced the most pronounced decreases in both age-standardized mortality rate (ASMR) and DALYs rates (ASDR). Specifically, the ASMR in these regions dropped from 6.8 (95% UI: 6.1–7.7) to 1.1 (0.9–1.3) per 100,000, with an EAPC of −6.17 (–6.46 to −5.87). In contrast, low SDI countries showed the least decline in both ASMR and ASDR. ASMR decreased modestly from 23.9 (21.0–28.7) to 17.8 (14.8–21.6) per 100,000 in low SDI regions, with an EAPC of −0.79 (–0.91 to −0.68). ASDR declined from 2,159.5 (1,895.7–2,591.4) to 1,732.3 (1,483.8–2,045.3) per 100,000, with an EAPC of −0.71 (–0.81 to −0.60). As of 2021, middle, low-middle, and low SDI regions continued to bear the highest burden of NE, reflected in elevated incidence and DALY rates (Table 1).

**Table 1:** Region-wise neonatal encephalopathy due to birth asphyxia and trauma burden in 2021.

Location	Sex	Deaths (95% UI), 1990		Deaths (95% UI), 2021			DALY (95% UI), 1990		DALY (95% UI), 2021		
		Case		Case			Case		Case		
						EAPC of ASMR (95%CI), 1990–2021					EAPC of ASDR (95%CI), 1990–2021
			ASMR (per100,000),		ASMR (per100,000),			ASDR (per100,000),		ASDR (per100,000),	
Global	Both	883,082.1 (809,080.1–1,004,097.1)	13.8 (12.7–15.7)	603,605.6 (511,190.6–725,270.8)	9.8 (8.3–11.7)	−1.24 (−1.36 to −1.11)	81,020,505.6 (74,272,388–92,120,587.4)	1,270.7 (1,164.6–1,443.7)	58,575,090.6 (50,032,890.5–68,835,375.5)	952.3 (819.3–1,105)	−1.11 (−1.23 to −0.99)
	Male	517,580.2 (462,704.9–594,591.7)	15.6 (14–18)	354,306.2 (293,053.9–422,742.4)	11.1 (9.2–13.2)	−1.2 (−1.33 to −1.07)	47,513,074.8 (42,609,788.8–54,699,632)	1,439.3 (1,291–1,657.6)	34,414,118.3 (28,910,398.8–40,569,208.2）	1,060.2 (889.8–1,252.6)	−1.08 (−1.2 to −0.96)
	Female	365,501.8 (324,486.1–422,333.3)	11.9 (10.5–13.7)	249,299.4 (207,717.8–297,288.9)	8.3 (6.9–9.9)	−1.28 (−1.41 to −1.15)	33,507,430.7 (29,736,634.2–38,645,726.5)	1,089.6 (967.9–1,256.8)	24,160,972.3 (20,445,156.7–28,526,985.7)	795.4 (670.5–942.3)	−1.16 (−1.28 to −1.04)
High SDI	Both	9,643.2 (9,090.1–10,301.5)	1.6 (1.5–1.7)	3,263.6 (2,896–3,592.9)	0.7 (0.6–0.7)	−2.59 (−2.69 to −2.49)	1,184,101.7 (1,052,167.3–1,394,334.1)	181.7 (164.8–206.8)	692,945.4 (578,891.6–810,162.3)	107.3 (95.8–119.8)	−1.73 (−1.82 to −1.64)
	Male	5,503 (5,069.3–6,026.4)	1.8 (1.6–1.9)	1,753.3 (1,550–1,954.4)	0.7 (0.6–0.8)	−2.73 (−2.84 to −2.63)	690,157.2 (605,254.7–809,602.9)	206 (184–236.4)	405,788 (335,332.7–478,567.5）	111.4 (95.6–127.2)	−1.75 (−1.85 to −1.66)
	Female	4,140.2 (3,871.9–4,491)	1.4 (1.3–1.5)	1,510.3 (1,355.5–1,642.3)	0.6 (0.6–0.7)	−2.41 (−2.5 to −2.31)	493,944.6 (438,984.6–577,308.4)	156.1 (141.6–176.7)	287,157.4 (243,605–332,606.9)	86.7 (76.6–98)	−1.69 (−1.78 to −1.6)
High-middle SDI	Both	59,663.9 (53,627.6–67,336.5)	6.8 (6.1–7.7)	6,141.8 (5,240.1–7,168.8)	1.1 (0.9–1.3)	−6.17 (−6.46 to −5.87)	5,828,345 (5,190,972.1–6,492,637.7)	653.6 (581.4–731.2)	1,283,112.4 (1,070,506.9–1,516,877.7)	176.7 (155.5–204.5)	−4.92 (−5.11 to −4.72)
	Male	35,057.5 (30,495.9–40,821.3)	7.6 (6.6–8.9)	3,530.7 (2,977.6–4,180.5)	1.2 (1–1.4)	−6.23 (−6.53 to −5.93)	3,439,434.5 (2,990,581–3,944,677)	738.1 (642.6–846)	770,517.7 (640,390–919,021.1）	180.7 (154.2–212.9)	−4.88 (−5.07 to −4.69)
	Female	24,606.4 (21,711–28,135.8)	5.9 (5.2–6.7)	2,611.2 (2,201.9–3,112.4)	1 (0.8–1.2)	−6.08 (−6.38 to −5.78)	2,388,910.5 (2,104,956.4–2,697,575.2)	561.3 (495.9–634)	512,594.6 (422,132.6–602,316.9)	133 (113.5–155.3)	−4.97 (−5.17 to −4.76)
Middle SDI	Both	230,185.3 (203,006.6–257,985.4)	11.5 (10.1–12.9)	62,255.8 (52,405.4–74,587.1)	4.1 (3.4–4.9)	−3.48 (−3.66 to −3.29)	21,311,358.7 (18,723,691.2–23,795,033.7)	1,067 (937.4–1,190.5)	7,157,674.9 (6,157,993.9–8,278,075.5)	464.8 (404.7–536.4)	−3.06 (−3.21 to −2.91)
	Male	137,751.1 (117,206.9–159,448.3)	13.2 (11.2–15.3)	37,212.7 (30,477.9–45,004.9)	4.7 (3.8–5.6)	−3.48 (−3.66 to −3.31)	12,747,081.4 (10,911,792.7–14,634,898.1)	1,224.3 (1,049.4–1,404.7)	4,279,266.5 (3,629,377.7–5,007,492.1）	495.4 (417.8–585.4)	−3.06 (−3.2 to−2.91)
	Female	92,434.2 (79,091.7–104,446.9)	9.6 (8.3–10.9)	25,043.1 (21,096.6–29,911.8)	3.4 (2.9–4.1)	−3.46 (−3.65 to −3.27)	8,564,277.3 (7,343,577.9–9,632,661.2)	895.6 (768.2–1,007.3)	2,878,408.4 (2,438,731.3–3,348,174.6)	359.2 (305.4–419.7)	−3.07 (−3.23 to −2.91)
Low-middle SDI	Both	330,761.6 (290,033.1–398,249.8)	17.9 (15.7–21.5)	225,205.6 (184,776.5–270,362.9)	12.1 (9.9–14.5)	−1.28 (−1.42 to −1.14)	29,913,901 (26,276,584.6–36,064,907.3)	1,618.3 (1,424.3–1,950.9)	21,150,947.2 (17,643,752.1–25,266,492.4)	1,170.4 (984.9–1,373.4)	−1.18 (−1.31 to −1.05)
	Male	191,559.7 (163,555.4–233,691.9)	20.1 (17.1–24.5)	129,485.7 (104,029.9–156,736.3)	13.4 (10.8–16.3)	−1.25 (−1.39 to −1.11)	17,327,659.6 (14,813,856.9–21,108,710)	1,818.4 (1,555.3–2,213.3)	12,175,317.8 (9,861,663.3–14,612,429.5）	1,261 (1,021.2–1,513.7)	−1.15 (−1.27 to −1.02)
	Female	139,201.9 (117,489.6–171,596.1)	15.5 (13.1–19.1)	95,719.9 (76,541.3–119,299.9)	10.6 (8.5–13.2)	−1.32 (−1.46 to −1.17)	12,586,241.4 (10,632,684.5–15,483,175.7)	1,405.4 (1,189.1–1,727.7)	8,975,629.4 (7,250,928.2–11,137,183.9)	991.5 (800.4–1,231.5)	−1.22 (−1.36 to −1.09)
Low SDI	Both	252,433.9 (221,890.8–302,642.2)	23.9 (21–28.7)	306,445.3 (254,816.5–372,292.4)	17.8 (14.8–21.6)	−0.79 (−0.91 to −0.68)	22,745,314.6 (19,992,946.6–27,264,218.1)	2,159.5 (1,895.7–2,591.4)	28,260,542.8 (23,675,059.8–34,105,504)	1,732.3 (1,483.8–2,045.3)	−0.71 (−0.81 to −0.6)
	Male	147,476.2 (125,689.1–180,294.7)	27.3 (23.2–33.4)	182,153 (147,907.7–225,155.3)	20.6 (16.7–25.4)	−0.74 (−0.87 to −0.6)	13,286,505.6 (11,341,251.6–16,243,162.9)	2,458.3 (2,096.7–3,010)	16,765,668 (13,732,992.8–20,586,532.7）	1,910.8 (1,568.8–2,340.2)	−0.65 (−0.77 to −0.54)
	Female	104,957.7 (87,944.9–129,244.7)	20.4 (17.1–25.2)	124,292.3 (102,400–149,930.7)	14.8 (12.2–17.9)	−0.88 (−0.98 to −0.78)	9,458,809 (7,921,124.3–11,653,222)	1,844.6 (1,542.6–2,275.7)	11,494,874.7 (9,544,778.2–13,775,869.5)	1,382.8 (1,150.8–1,655.8)	−0.78 (−0.86 to −0.7)
Andean Latin America	Both	5,429.7 (4,477.5–6,495.6)	9.7 (8–11.6)	1,893.5 (1,380.3–2,443.7)	3.2 (2.3–4.1)	−3.09 (−3.29 to −2.9)	499,197.6 (409,632.9–595,237.4)	895 (734.7–1,065.2)	215,974.3 (170,130.4–271,026.3)	435.7 (348.6–543.9)	−2.6 (−2.75 to −2.45)
	Male	3,064.6 (2,361.1–3,747.5)	10.7 (8.2–13.1)	1,044.9 (739.7–1,423.1)	3.4 (2.4–4.7)	−3.14 (−3.36 to −2.91)	281,877.3 (217,433.6–343,168.8)	991.8 (768.7–1,208.9)	119,320.2 (91,594.7–153,753.7）	383.4 (293.5–496)	−2.64 (−2.81 to −2.48)
	Female	2,365.2 (1,819.2–3,020.4)	8.6 (6.6–11)	848.6 (594.3–1,127.7)	2.9 (2–3.9)	−3.05 (−3.23 to −2.87)	217,320.3 (167,860.2–275,943)	794.4 (615.1–1,004.6)	96,654.1 (72,609.8–123,073.3)	324.2 (242.1–415.4)	−2.55 (−2.7 to −2.41)
Australasia	Both	193.8 (180.6–208.7)	1.3 (1.2–1.4)	129.3 (107.3–156.6)	0.8 (0.6–0.9)	−1.01 (1.61 to −0.41)	23,777.4 (21,163.8–26,834.5)	146.3 (132.5–162.5)	19,756.2 (16,818.6–23,325.9)	116.3 (105.2–128.3)	−0.92 (−1.38 to −0.46)
	Male	104.4 (95.3–114.6)	1.3 (1.2–1.5)	73.4 (60.1–89.6)	0.8 (0.7–1)	−0.89 (−1.43 to −0.34)	13,058.7 (11,441.6–14,709.7)	156.8 (139.2–174.8)	11,334.6 (9,607.8–13,398.4）	107.8 (91.8–126.2)	−0.81 (−1.21 to −0.4)
	Female	89.4 (82.6–97.4)	1.2 (1.1–1.3)	55.9 (46.6–67.5)	0.7 (0.6–0.8)	−1.16 (−1.85 to −0.46)	10,718.8 (9,535.1–12,178.2)	135.4 (122.3–151.5)	8,421.5 (7,120.5–9,905.2)	83.5 (71.4–98.2)	−1.06 (−1.61 to −0.51)
Caribbean	Both	4,044.3 (3,420.8–4,802.2)	9.4 (7.9–11.1)	3,063.7 (2,331.6–4,052.8)	8 (6.1–10.6)	−0.32 (−0.44 to −0.19)	380,370.9 (324,076.5–450,596.5)	888.5 (756.8–1,051.1)	308,417.7 (241,738.5–398,520.6)	800.4 (624.6–1,032.2)	−0.21 (−0.33 to −0.09)
	Male	2,411.1 (1,885.3–2,966.6)	10.9 (8.6–13.5)	1,779.5 (1,210.7–2,494.7)	9.1 (6.2–12.8)	−0.38 (−0.55 to −0.21)	228,118.2 (180,845.5–278,600.9)	1,045.7 (831.6–1,277.5)	182,520.2 (130,740.5–246,605.5）	917.8 (652.7–1,246.3)	−0.25 (−0.41 to −0.09)
	Female	1,633.2 (1,312.6–2,028.8)	7.7 (6.2–9.6)	1,284.2 (892.5–1,809.4)	6.9 (4.8–9.7)	−0.22 (−0.29 to −0.15)	152,252.7 (122,738.2–187,823.9)	725.3 (584.5–893.7)	125,897.5 (90,146.1–171,292.3)	664.5 (473.6–910)	−0.15 (−0.22 to −0.09)
Central Asia	Both	11,408.2 (10,302.4–12,572.1)	12.1 (10.9–13.3)	4,565.8 (3,842–5,421.4)	4.6 (3.9–5.5)	−3.06 (−3.69 to −2.42)	1,050,229.1 (948,674.9–1,156,367.8)	1,119.6 (1,010.1–1,233)	480,540.5 (409,925.5–556,469.7)	526.8 (453.3–612.4)	−2.67 (−3.24 to −2.1)
	Male	6,952.1 (6,227–7,771.1)	14.4 (12.9–16.1)	2,843.3 (2,360.2–3,410.4)	5.6 (4.6–6.7)	−3.03 (−3.68 to −2.37)	641,823.4 (575,655.5–715,162.1)	1,337.5 (1,199.2–1,489.3)	306,391.3 (259,970.6–360,165.4）	603.3 (512.2–708.9)	−2.57 (−3.15 to −1.99)
	Female	4,456.1 (3,943.6–4,936.8)	9.7 (8.6–10.7)	1,722.5 (1,454.6–2,055.3)	3.6 (3.1–4.4)	−3.12 (−3.72 to −2.52)	408,405.7 (363,424.8–451,261.4)	891.4 (794–985)	174,149.1 (149,361.5–204,485.6)	367.4 (315.1–431.7)	−2.85 (−3.41 to −2.3)
Central Europe	Both	2,659.9 (2,424.6–2,867.5)	3.2 (2.9–3.5)	254.8 (215.1–298)	0.5 (0.4–0.6)	−6.43 (−6.67 to −6.19)	298,467.2 (266,028.4–336,822.2)	338.4 (304.3–373.7)	79,573.7 (64,235.8–95,803.9)	106.8 (90.9–122.7)	−4.31 (−4.51 to −4.11)
	Male	1,568.6 (1,410.1–1,707.4)	3.7 (3.3–4)	145.9 (120.2–173.6)	0.6 (0.5–0.7)	−6.57 (−6.82 to −6.31)	179,704.7 (159,410.3–204,243)	397.6 (356.2–443.3)	51,332.1 (40,897.4–61,949.6）	125.9 (103.9–148)	−4.14 (−4.34 to −3.94)
	Female	1,091.3 (960.4–1,209.4)	2.7 (2.4–3)	108.9 (92.4–127.3)	0.4 (0.4–0.5)	−6.24 (−6.48 to −6.01)	118,762.5 (103,041.5–134,796.5)	276.8 (242.5–310.1)	28,241.6 (23,323.8–33,595.4)	75.7 (64.4–87.8)	−4.59 (−4.79 to −4.39)
Central Latin America	Both	18,472.6 (17,246.1–19,960.6)	7.7 (7.2–8.3)	4,546.3 (3,596.8–5,762.8)	2.4 (1.9–3.1)	−3.45 (−3.64 to −3.25)	1,769,588.8 (1,641,200–1,945,218.7)	752.9 (697–834.9)	619,820 (517,686.7–742,075.9)	342.6 (291.2–396.7)	−2.77 (−2.96 to −2.59)
	Male	10,710.8 (9,911.3–11,685.8)	8.8 (8.1–9.6)	2,626.6 (2,020.3–3,396.6)	2.7 (2.1–3.5)	−3.52 (−3.74 to −3.29)	1,030,355 (950,451.5–1,145,053.2)	863.7 (794.7–962.6)	363,675.3 (299,780.4–440,597.1）	349.7 (285.6–426.3)	−2.79 (−3 to −2.58)
	Female	7,761.8 (7,231.2–8,368.3)	6.6 (6.1–7.1)	1,919.7 (1,553.2–2,380.4)	2.1 (1.7–2.6)	−3.35 (−3.52 to −3.18)	739,233.8 (684,261.1–804,670.5)	638.8 (589–700.8)	256,144.7 (215,943–301,397.6)	252.4 (212.9–301.1)	−2.75 (−2.9 to −2.59)
Central Sub-Saharan Africa	Both	30,702.3 (23,438.1–38,413.8)	25 (19.1–31.4)	37,710.5 (29,788.1–47,900.4)	17.7 (13.9–22.4)	−0.67 (−0.92 to −0.42)	2,766,035.5 (2,112,142–3,458,551.4)	2,257.9 (1,720.8–2,831.2)	3,480,237.7 (2,767,823.3–4,385,334.9)	1,811.7 (1,456.5–2,249.8)	−0.59 (−0.82 to −0.35)
	Male	17,196.8 (12,951–21,534.9)	27.5 (20.6–34.4)	22,336 (16,999.4–28,524.5)	20.5 (15.6–26.2)	−0.42 (−0.7 to −0.15)	1,549,134.9 (1,166,837.4–1,941,102)	2,475.9 (1,860.2–3,105.6)	2,055,270.1 (1,575,363.5–2,608,067.3）	1,901.8 (1,461.8–2,408.7)	−0.35 (−0.61 to −0.09)
	Female	13,505.5 (8,055.1–17,777)	22.5 (13.4–29.7)	15,374.5 (11,274.6–19,730)	14.7 (10.8–18.9)	−1 (−1.22 to −0.78)	1,216,900.5 (729,018.2–1,600,716.3)	2,030.4 (1,216.4–2,678.4)	1,424,967.5 (1,054,174.5–1,815,270.9)	1,376.7 (1,021.7–1,749.9)	−0.9 (−1.1 to −0.7)
East Asia	Both	140,405 (115,423.3–166,876.3)	12.2 (10.1–14.5)	9,724.3 (8,006.3–11,756.9)	1.7 (1.4–2.1)	−6.71 (−7.22 to −6.19)	13,083,122.9 (10,743,691.5–15,465,180.9)	1,136.8 (933.4–1,343.6)	1,904,282.5 (1,598,699.2–2,279,342.8)	269.9 (231.2–315.3)	−5.64 (−6.04 to −5.24)
	Male	82,612.2 (64,719.6–102,384.1)	13.5 (10.6–16.7)	5,747.1 (4,618.6–7,131.1)	1.9 (1.5–2.4)	−6.66 (−7.16 to −6.15)	7,704,258.2 (6,106,176.6–9,495,983.9)	1,253.2 (992.9–1,544.7)	1,136,857.7 (941,117.7–1,371,636.2）	260.8 (220.3–306.3)	−5.55 (−5.94 to −5.16)
	Female	57,792.8 (46,085.9–69,736.4)	10.8 (8.6–13.1)	3,977.1 (3,120–4,809.1)	1.5 (1.2–1.9)	−6.78 (−7.31 to −6.26)	5,378,864.7 (4,290,891.5–6,442,229.2)	1,003.5 (800.7–1,201.3)	767,424.8 (636,946.8–919,498.1)	199.9 (168.4–233.2)	−5.76 (−6.18 to −5.35)
Eastern Europe	Both	8,757.1 (8,372.2–9,115.1)	6.1 (5.8–6.3)	710.1 (632–791.7)	0.8 (0.7–0.9)	−6.52 (−6.87 to −6.17)	877,110.9 (822,257.2–955,653.1)	589 (555.9–626)	144,350.5 (120,978–168,653.1)	129.9 (115.6–145.7)	−5.37 (−5.58 to −5.15)
	Male	5,230.9 (4,935.1–5,517.4)	7.1 (6.7–7.5)	411 (360.9–463)	0.9 (0.8–1)	−6.71 (−7.06 to −6.37)	529,860.3 (490,568–577,948.5)	693.7 (647.2–744.2)	89,756.5 (74,582.6–106,206）	143.4 (124–163.9)	−5.34 (−5.53 to −5.15)
	Female	3,526.2 (3,359–3,718.4)	5 (4.8–5.3)	299.1 (267.5–333.5)	0.7 (0.6–0.8)	−6.26 (−6.62 to −5.9)	347,250.6 (326,993.4–377,780.8)	479.3 (454.7–512.5)	54,594 (46,356–63,128.1)	92.8 (82–103.6)	−5.39 (−5.64 to −5.14)
Eastern Sub−Saharan Africa	Both	99,536.2 (85,146–120,061.3)	23.4 (20–28.3)	100,837.4 (80,791.9–125,859.4)	15.4 (12.3–19.2)	−1.27 (−1.45 to −1.09)	8,979,620.6 (7,699,136.3–10,843,665.3)	2,112.7 (1,811.5–2,560.8)	9,478,336.7 (7,653,085.3–11,650,539.5)	1,546.9 (1,281.8–1,863.6)	−1.11 (−1.26 to −0.96)
	Male	59,425 (48,548.9–73,276.3)	27.2 (22.2–33.6)	61,994.7 (48,378–80,308.5)	18.5 (14.4–24)	−1.18 (−1.37 to −0.98)	5,356,970.7 (4,380,200.3–6,596,101.1)	2,460 (2,010.5–3,033.8)	5,794,930.1 (4,576,097.8–7,448,144.2）	1,755.7 (1,392.3–2,247.4)	−1.04 (−1.21 to −0.87)
	Female	40,111.1 (33,372–49,360.8)	19.3 (16–23.8)	38,842.7 (30,514.2–49,252.9)	12.1 (9.5–15.4)	−1.4 (−1.56 to −1.24)	3,622,649.9 (3,009,564.6–4,452,206.4)	1,748.3 (1,448.3–2,149.8)	3,683,406.5 (2,915,484.1–4,600,831)	1,169.7 (932.2–1,455.9)	−1.23 (−1.35 to −1.1)
High-income Asia Pacific	Both	906.6 (808.7–1,026.9)	1 (0.8–1.1)	137.7 (122.7–152.5)	0.2 (0.2–0.3)	−4.09 (−4.38 to −3.79)	165,835.6 (128,184.8–228,977.7)	135.6 (111.6–173.5)	95,889.1 (72,456.4–120,659.5)	79.1 (64.3–95.4)	−1.74 (−1.97 to −1.51)
	Male	505.6 (446.3–583.3)	1 (0.9–1.2)	69.3 (60.7–78.3)	0.2 (0.2–0.3)	−4.21 (−4.5 to −3.91)	96,251.9 (73,232.9–135,604.7)	152 (122–199.1)	57,626.9 (42,892–73,415.5）	85.7 (67.3–105.2)	−1.6 (−1.83 to −1.37)
	Female	401 (340.3–470.3)	0.9 (0.7–1)	68.4 (60.6–76.1)	0.2 (0.2–0.3)	−3.95 (−4.27 to −3.63)	69,583.7 (54,528.1–94,254.9)	118.4 (96.4–146.8)	38,262.2 (29,351.7–47,902.3)	61.8 (50.3–74.2)	−1.95 (−2.19 to −1.7)
High-income North America	Both	2,649 (2,576.3–2,725.8)	1.2 (1.2–1.2)	1,593.9 (1,418.6–1,772.3)	0.8 (0.7–0.9)	−0.93 (−1.05 to −0.81)	327,144 (289,688.4–393,296.8)	141.5 (127.4–166)	253,087.2 (217,666.5–288,531.3)	115.6 (106.1–126.5)	−0.71 (−0.8 to −0.62)
	Male	1,498.3 (1,448.2–1,551.9)	1.3 (1.3–1.4)	859.3 (757.6–971.1)	0.9 (0.8–1)	−0.98 (−1.12 to −0.85)	190,227.7 (165,831.7–231,866.1)	161 (142.6–192.2)	144,277.7 (122,381–165,996.2）	117.5 (102.7–133)	−0.75 (−0.85 to −0.66)
	Female	1,150.6 (1,112.6–1,188)	1.1 (1–1.1)	734.6 (655.8–817.5)	0.8 (0.7–0.9)	−0.86 (−0.99 to −0.74)	136,916.3 (122,739.9–161,589.5)	121.1 (110.5–139.7)	108,809.5 (94,493.6–123,409.9)	94.3 (84.2–105)	−0.65 (−0.74 to −0.55)
North Africa and Middle East	Both	34,054.8 (28,916.4–42,412.4)	6.5 (5.5–8.1)	16,645.3 (13,107.2–20,435.5)	2.9 (2.3–3.6)	−2.8 (−2.93 to −2.67)	3,125,160.8 (2,646,260.3–3,882,448.4)	601.7 (509.6–746.9)	1,748,769.7 (1,450,210.3–2,127,320.2)	318.4 (264.7–381.8)	−2.46 (−2.58 to −2.34)
	Male	19,980.3 (16,009.9–26,088.5)	7.4 (5.9–9.7)	9,716.4 (7,443.4–11,990.7)	3.3 (2.5–4.1)	−2.84 (−2.97 to −2.71)	1,833,183.8 (1,467,571.8–2,387,646.8)	686 (551.2–890.3)	1,018,621.9 (815,638.4–1,238,408.3）	338.9 (270–412.9)	−2.51 (−2.63 to −2.38)
	Female	14,074.5 (11,361.1–17,958.8)	5.5 (4.5–7.1)	6,928.9 (5,152–8,930)	2.5 (1.9–3.2)	−2.74 (−2.87 to −2.62)	1,291,976.9 (1,045,278.4–1,641,590.2)	512.3 (414.8–649.9)	730,147.7 (566,442.7–909,288.6)	258.5 (199.6–322.7)	−2.39 (−2.51 to −2.28)
Oceania	Both	645.5 (495.2–837.7)	6 (4.6–7.8)	1,011.8 (714.4–1,384.3)	4.9 (3.5–6.8)	−0.62 (−0.74 to −0.49)	59,281.4 (45,854.2–76,612.7)	556.6 (432–717.2)	95,677.8 (69,131.3–128,535)	477.7 (345.2–642.6)	−0.51 (−0.65 to −0.37)
	Male	342.1 (244.2–491.5)	6.1 (4.4–8.8)	507.2 (313.9–799.6)	4.7 (2.9–7.5)	−0.86 (−0.97 to −0.75)	31,541.9 (22,731–44,851.4)	569.3 (412–804.7)	48,553.5 (31,153.1–74,721.4）	462.8 (299.3–705.8)	−0.72 (−0.84 to −0.6)
	Female	303.3 (212–414.7)	5.9 (4.1–8)	504.6 (316.6–724.7)	5.2 (3.2–7.4)	−0.36 (−0.54 to −0.19)	27,739.5 (19,597.5–37,805.2)	542.8 (385.4–737.8)	47,124.3 (30,086.1–67,015.8)	487.7 (312.9–690.7)	−0.28 (−0.47 to −0.1)
South Asia	Both	311,442.1 (258,810.6–411,799.8)	19 (15.7–25.1)	195,871.6 (154,828.9–254,921.2)	12.9 (10.2–16.8)	−1.25 (−1.36 to −1.14)	28,152,333.2 (23,394,123.9–37,166,964.2)	1,715.7 (1,425.5–2,264.5)	18,411,973.3 (14,634,166–23,790,913.6)	1,226.5 (985.6–1,579.7)	−1.16 (−1.26 to −1.06)
	Male	180,488.6 (144,456.1–242,140.2)	21.2 (17–28.4)	112,959.2 (87,362.6–145,773.8)	14.3 (11.1–18.5)	−1.22 (−1.32 to −1.11)	16,326,654.8 (13,073,175–21,902,935.1)	1,921.2 (1,539.2–2,577.9)	10,662,509.5 (8,377,689.4–13,544,751.7）	1,338.4 (1,050.1–1,705.2)	−1.12 (−1.21 to −1.02)
	Female	130,953.5 (105,958.4–175,853.8)	16.5 (13.4–22.2)	82,912.4 (63,392.1–114,842.3)	11.5 (8.8–15.9)	−1.3 (−1.42 to −1.18)	11,825,678.4 (9,561,512.2–15,868,605.6)	1,494.8 (1,208.2–2,004.6)	7,749,463.8 (6,040,658.2–10,604,982.7)	1,061.7 (825.9–1,456.8)	−1.22 (−1.33 to −1.11)
Southeast Asia	Both	52,902.8 (39,416.4–64,777.8)	8.9 (6.7–10.9)	24,371.7 (17,335.4–30,366.1)	4.5 (3.2–5.6)	−2.2 (−2.34 to −2.06)	4,953,401.7 (3,734,099.7–5,997,030.5)	842.1 (635.8–1,019.1)	2,627,366.9 (1,999,404.2–3,174,278.9)	503.9 (372.1–606.9)	−1.91 (−2.04 to −1.79)
	Male	32,255.9 (21,073.5–41,778.5)	10.6 (6.9–13.7)	14,853 (9,337.9–19,124.5)	5.3 (3.4–6.9)	−2.14 (−2.26 to −2.02)	3,008,800.8 (2,037,483.5–3,863,712.4)	992.6 (678.6–1,271.8)	1,588,466.2 (1,087,528.7–1,979,587.9）	550.8 (371.3–689.7)	−1.85 (−1.96 to −1.75)
	Female	20,646.9 (16,276.8–24,721)	7.2 (5.7–8.6)	9,518.6 (7,308.3–11,580)	3.6 (2.8–4.4)	−2.3 (−2.49 to −2.11)	1,944,601 (1,560,222.4–2,320,708.6)	682.1 (547.5–813.1)	1,038,900.7 (843,448.3–1,235,194)	380.3 (307.2–453.1)	−2.01 (−2.17 to −1.84)
Southern Latin America	Both	1,961.6 (1,785.1–2,140.9)	3.9 (3.5–4.2)	319.1 (249.7–399.5)	0.9 (0.7–1.1)	−4.66 (−4.89 to −4.42)	206,047.8 (185,669.9–230,629.1)	406.8 (366.5–455.4)	73,264.6 (59,602.1–87,751.2)	160.6 (140–182.6)	−3.22 (−3.46 to −2.99)
	Male	1,101.9 (982.5–1,226.8)	4.3 (3.8–4.7)	180.4 (136–227.6)	0.9 (0.7–1.2)	−4.67 (−4.88 to −4.47)	116,831.6 (102,943.9–132,723.8)	454.1 (399.9–516.7)	43,026.2 (34,928.1–52,257.9）	168.2 (138.6–202.1)	−3.15 (−3.36 to −2.94)
	Female	859.8 (775.1–959.1)	3.5 (3.1–3.9)	138.7 (107.9–176.8)	0.8 (0.6–1)	−4.64 (−4.92 to −4.35)	89,216.3 (79,897.1–100,355.9)	358.1 (320.6–402.8)	30,238.3 (24,739.1–36,321.5)	122.4 (100.1–145.7)	−3.32 (−3.59 to −3.05)
Southern Sub-Saharan Africa	Both	9,251.2 (7,811–11,025.7)	11.9 (10.1–14.2)	6,469.2 (4,997.5–8,456.4)	8.3 (6.4–10.8)	−1.15 (−1.29 to −1.02)	846,956.4 (716,256.3–1,000,972.5)	1,096.7 (926.6–1,296)	635,226 (503,824.9–811,682.7)	744.3 (597.1–923.4)	−0.97 (−1.1 to −0.84)
	Male	5,411.7 (4,439.9–6,638)	13.7 (11.2–16.8)	3,846.1 (2,838.9–5,097.8)	9.7 (7.2–12.8)	−1.09 (−1.22 to −0.96)	494,505.7 (408,237.8–603,303.8)	1,257.3 (1,037.6–1,530.2)	375,692.4 (289,070.9–491,291.7）	942 (723.5–1,232.9)	−0.91 (−1.04 to −0.78)
	Female	3,839.6 (3,069.6–4,715.2)	10.1 (8.1–12.4)	2,623.1 (1,949.9–3,453.7)	6.8 (5.1–9)	−1.24 (−1.38 to −1.1)	352,450.7 (281,266.7–433,583.6)	930 (741.1–1,142.9)	259,533.6 (201,704.1–337,920)	669.1 (518.4–872.5)	−1.05 (−1.18 to −0.92)
Tropical Latin America	Both	12,030.8 (10,782–13,234.9)	7.5 (6.7–8.2)	4,479 (3,613.8–5,534.7)	2.7 (2.2–3.3)	−2.95 (−3.21 to −2.69)	1,116,999.2 (1,002,967.9–1,233,349.3)	692.9 (622.2–764.7)	485,943.1 (403,748.2–580,716.5)	305.3 (254.5–366.5)	−2.64 (−2.88 to −2.41)
	Male	6,997.2 (6,217.3–7,744.1)	8.5 (7.6–9.4)	2,494.3 (1,966.4–3,132.2)	2.9 (2.3–3.7)	−3.08 (−3.36 to −2.79)	646,138.2 (575,539.4–713,542.3)	785.8 (700–867.9)	266,873.2 (218,041.7–321,439.0）	303.3 (247.3–368.1)	−2.77 (−3.03 to −2.52)
	Female	5,033.6 (4,564.1–5,556)	6.4 (5.8–7)	1,984.7 (1,606.2–2,421.4)	2.5 (2–3)	−2.79 (−3.03 to −2.55)	470,861.1 (425,206.9–520,726.1)	596 (538.4–659.1)	219,069.9 (183,036.9–260,124)	257 (213.8–306.8)	−2.48 (−2.69 to −2.26)
Western Europe	Both	3,283.3 (3,189.8–3,387.5)	1.5 (1.4–1.5)	1,287.4 (1,116–1,454.6)	0.7 (0.6–0.7)	−2.19 (−2.4 to −1.98)	421,594.2 (376,823.5–486,920.7)	167.8 (155–185.5)	251,161.6 (211,647.5–292,078.7)	99.6 (89.7–109.9)	−1.54 (−1.71 to −1.37)
	Male	1,870.7 (1,811.3–1,930.9)	1.6 (1.6–1.7)	678.8 (575.7–779.7)	0.7 (0.6–0.8)	−2.41 (−2.63 to −2.18)	244,688.9 (217,326.9–281,844.7)	189.9 (174.4–210.8)	141,891.7 (118,051.8–166,571.6）	101.9 (88.3–116.9)	−1.65 (−1.83 to −1.48)
	Female	1,412.6 (1,365.7–1,462)	1.3 (1.3–1.3)	608.6 (531.7–682.2)	0.6 (0.6–0.7)	−1.93 (−2.12 to −1.74)	176,905.3 (159,229–202,186.1)	144.7 (134.4–159.1)	109,269.9 (93,715.5–126,515.9)	84.4 (74.6–95)	−1.39 (−1.55 to −1.23)
Western Sub-Saharan Africa	Both	132,345.3 (115,019.7–160,700.9)	31.1 (27–37.7)	187,983.4 (152,955.3–224,574.3)	22.1 (18–26.4)	−0.95 (−1.06 to −0.84)	11,918,230.4 (10,352,323.5–14,479,177.4)	2,799.8 (2,433.2–3,397.5)	17,165,441.6 (14,001,956.8–20,467,520.5)	2,154.6 (1,778.2–2,501.6)	−0.89 (−0.99 to −0.8)
	Male	77,851.3 (65,542.9–94,907.7)	35.8 (30.2–43.6)	109,139.9 (86,191.1–133,192.8)	25.1 (19.8–30.6)	−1.02 (−1.13 to −0.91)	7,009,088.2 (5,901,229.5–8,545,376.1)	3,226.5 (2,718.5–3,928.6)	9,955,190.9 (7,897,832.5–12,124,235.2）	2,309.6 (1,836.4–2,811.1)	−0.96 (−1.06 to −0.87)
	Female	54,493.9 (46,115.9–65,733.1)	26.1 (22.1–31.5)	78,843.5 (64,050.5–93,928.6)	19 (15.4–22.6)	−0.85 (−0.96 to −0.75)	4,909,142.2 (4,157,843.3–5,920,569.3)	2,355.3 (1,995–2,839.7)	7,210,250.7 (5,863,400.8–8,587,500.9)	1,748.2 (1,422.1–2,082)	−0.8 (−0.89 to −0.7)

ASMR, age-standardised mortality rate; ASDR, age-standardised DALYs rate.

Regionally, from 1990 to 2021, the age-standardized mortality rate (ASMR) of NE caused by asphyxia and trauma declined overall. East Asia showed the most substantial decline, with ASMR decreasing from 12.2 (95% UI: 10.1–14.5) to 1.7 (1.4–2.1) per 100,000 (EAPC: −6.71 [–7.22 to −6.19]). Eastern and Central Europe also showed substantial declines, with ASMRs decreasing from 6.1 (5.8–6.3) to 0.8 (0.7–0.9) and from 3.2 (2.9–3.5) to 0.5 (0.4–0.6), respectively. The Caribbean exhibited the minimal improvement, with ASMR decreasing only slightly from 9.4 (7.9–11.1) to 8.0 (6.1–10.6) (EAPC −0.32 [–0.44 to −0.19]). Oceania and Central Sub-Saharan Africa exhibited modest declines. By 2021, ASMR remained high in Western (22.1 [18.0–26.4]), Eastern (15.4 [12.3–19.2]), and Central (17.7 [13.9–22.4]) Sub-Saharan Africa. In high-burden regions, male neonatal mortality was consistently higher than female (Table 1).

Similarly, the age-standardized DALY rate (ASDR) declined globally. East Asia again showed the greatest reduction, from 1,136.8 (933.4–1,343.6) to 269.9 (231.2–315.3) per 100,000 (EAPC: −5.64 [–6.04 to −5.24]). Eastern and Central Europe also had considerable decreases. The Caribbean demonstrated the least progress, with ASDR declining only slightly from 888.5 (756.8–1,051.1) to 800.4 (624.6–1,032.2; EAPC: −0.21 [–0.33 to −0.09]). In 2021, the highest ASDRs persisted in Western (2,154.6 [1,778.2–2,501.6]), Eastern (1,546.9 [1,281.8–1,863.6]), and Central (1,811.7 [1,456.5–2,249.8]) Sub-Saharan Africa. Males experienced significantly higher DALYs than females in high-burden regions (Table 1).

### 3. Country-level trends in age-standardized incidence (ASIR), mortality (ASMR), DALY (ASDR), and prevalence rates (ASPR) of neonatal encephalopathy

**Figure 2:**
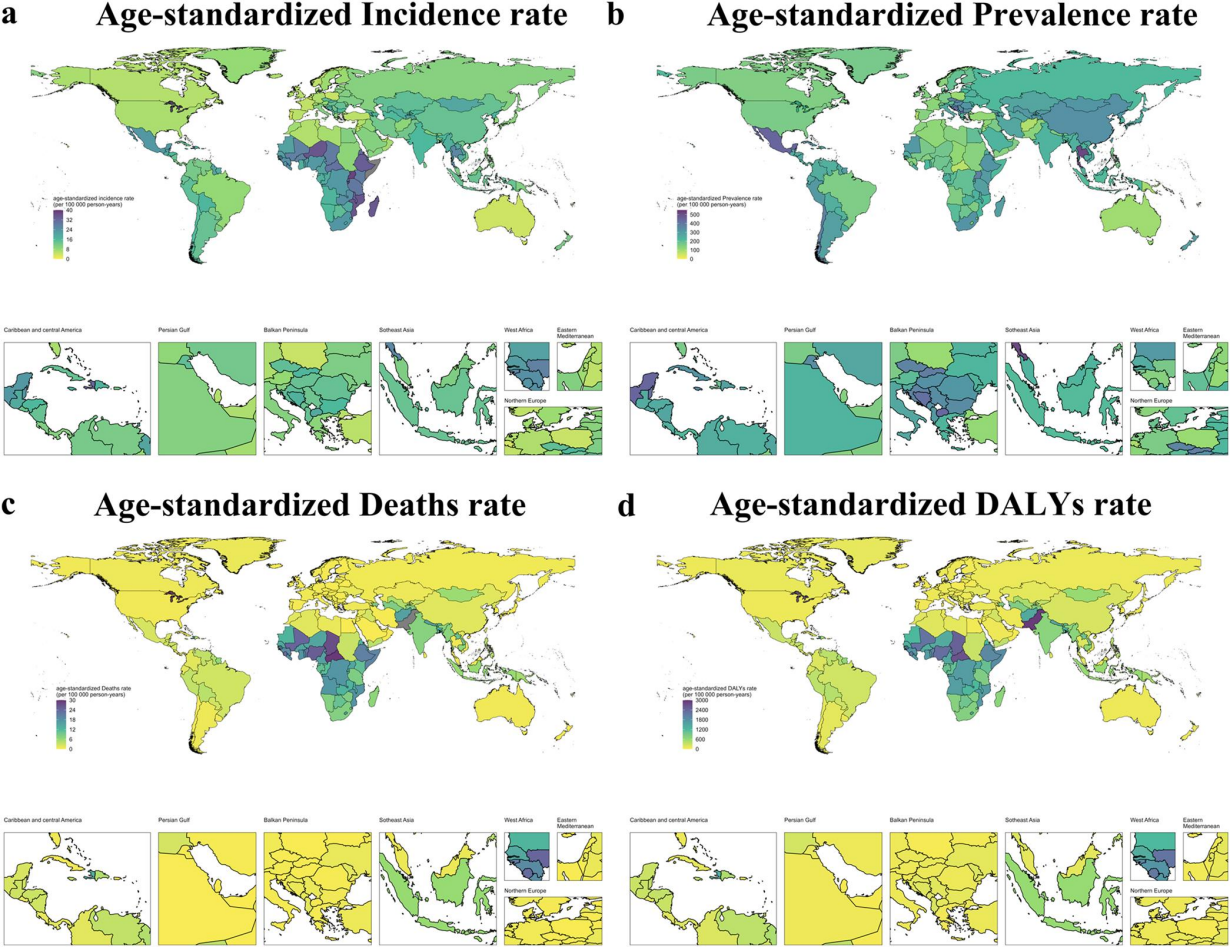
Geographical distribution of age-standardised rates of neonatal encephalopathy due to birth asphyxia and trauma in 2021 **(a)** ASIR in 2021; **(b)** ASPR in 2021; **(c)** ASMR in 2021; **(d)** ASDR in 2021.

In 2021, India, Nigeria, and China reported the highest number of new NE, with 169,064.4 (95% UI: 159,957.6–178,943.4), 90,497.7 (85,073.5–96,088.4), and 74,198.8 (70,060.5–78,261.4) cases, respectively. Nigeria had the most NE-related deaths at 100,304.6 (78,778.0–123,896.3), followed by Pakistan with 96,081.9 (73,472.3–119,862.7) and India with 77,652.0 (53,706.2–126,852.3) (Supplementary Appendix 4). Somalia (56.1 [52.7–59.4]), Burundi (39.6 [37.2–41.8]), and Uganda (38.3 [36.2–40.5]) had the highest age-standardized incidence rates (ASIR) per 100,000, while Portugal (3.2 [3.0–3.4]), Belgium (3.4 [3.2–3.6]), and Australia (3.9 [3.7–4.2]) had the lowest (Supplementary Appendix 4; Figure 2a).

The highest age-standardized prevalence rates(ASPR) per 100,000 were in Thailand (517 [452.9–590.2]), Mexico (447.5 [389.0–507.1]), and North Macedonia (442.9 [369.1–480.4]). and the lowest in the Central African Republic (75.4 [56.6–98.7]), Portugal (82.2 [73.0–93.2]), and Afghanistan (82.9 [66.5–101.2]) (Supplementary Appendix 4; Figure 2b) (Supplementary Appendix 4 Figure 2b). Pakistan had the highest age-standardized mortality rate (ASMR) at 32.2 (24.6–40.2) per 100,000. The lowest ASMRs were in Slovenia and Andorra (both 0.1 [0.1–0.2]) and the United Arab Emirates (0.2 [0.1–0.2]) (Supplementary Appendix 4; Figure 2c). For age-standardized DALY rates (ASDR), Pakistan also ranked highest at 2,928.0 (2,245.0–3,644.7) per 100,000, followed by the Central African Republic (2,687.0 [1,995.2–3,528.5]) and South Sudan (2,660.3 [1,788.8–3,713.3]). The lowest ASDRs were in Andorra (43.8 [32.2–56.6]), Poland (46.1 [37.3–55.2]), and Qatar (46.9 [35.7–59.7]) (Supplementary Appendix 4; Figure 2d).

**Figure 3:**
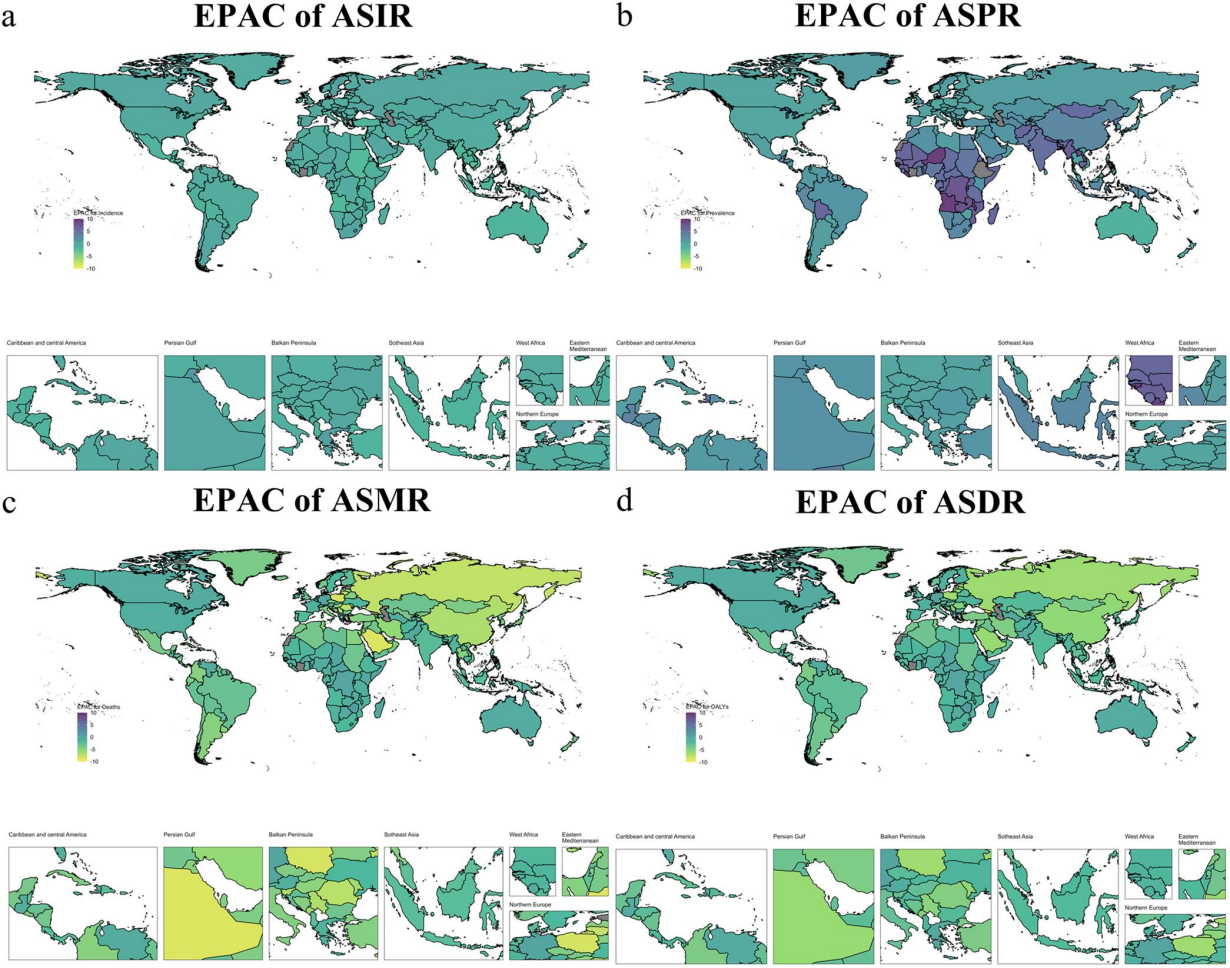
Geographical distribution of EPAC of neonatal encephalopathy due to birth asphyxia and trauma in 2021 **(a)** changes in percentage of ASIR in smoking(1990-2021);**(b)** changes in percentage of ASMR in smoking (1990-2021); **(c)** changes in percentage of ASDR in smoking (1990-2021); **(d)** changes in percentage of ASPR in smoking (1990-2021).

Between 1990 and 2021, the age-standardized incidence rate (ASIR) of NE decreased in 177 of 204 countries and territories. Timor-Leste showed the largest decline, from 41.5 (95% CI: 39.2–44.0) to 20.2 (19.0–21.3) per 100,000 (EAPC: −2.77 [–3.08 to −2.46]). Significant decreases also occurred in Equatorial Guinea (34.25 [32.4–36.3] to 17.9 [16.9–19.0]; EAPC: −2.57 [–2.98 to −2.17]) and Cambodia (38.6 [36.4–41.0] to 19.4 [18.3–20.6]; EAPC: −2.49 [–2.71 to −2.26]). In contrast, Kuwait experienced the largest ASIR increase, from 10.7 (10.1–11.4) to 14.7 (13.9–15.5; EAPC: 1.25 [0.77 to 1.73]). American Samoa and Northern Mariana Islands also showed increasing trends (Supplementary Appendix 5; Figure 3a).

Similarly, the age-standardized mortality rate (ASMR) declined in 198 countries. Georgia had the most pronounced decrease, from 21.3 (18.0–24.9) to 1.2 (1.0–1.6) per 100,000 (EAPC: −10.34 [–11.15 to −9.53]). Estonia and Saudi Arabia also showed substantial declines. The largest ASMR increase occurred in Taiwan (Province of China), from 0.2 (0.19–0.24) to 0.36 (0.3–0.4; EAPC: 3.3 [2.77 to 3.82]). Rises were also observed in Dominica and South Sudan (Supplementary Appendix 6; Figure 3b). (Supplementary Appendix 6; Figure 3b).

Between 1990 and 2021, age-standardized DALYs due to neonatal encephalopathy from birth asphyxia and trauma declined consistently across 197 countries and territories. Georgia showed the steepest decrease, from 1954.7 (95% CI: 1664.0–2277.5) to 184.4 (152.4–221.8) per 100,000 (EAPC: −9.08 [–9.82 to −8.34]). Estonia and Poland also experienced substantial declines. In contrast, Taiwan (Province of China) reported the largest increase, from 64.0 (48.2–85.1) to 82.0 (67.0–99.9) per 100,000 (EAPC: 1.55 [1.24–1.86]). Rises were also observed in Dominica and South Sudan (Supplementary Appendix 7; Figure 3c).

Over the same period, age-standardized prevalence increased across 184 countries and territories. Ethiopia recorded the greatest rise, from 12.0 (4.8–24.3) to 325.4 (256.8–406.2) per 100,000 (EAPC: 12.23 [11.97–12.48]). Rwanda and Equatorial Guinea also showed marked increases. Australia had the most significant decline, from 128.7 (108.3–151.3) to 99.9 (88.0–114.3) per 100,000 (EAPC: −1.06 [–1.50 to −0.61]). Portugal and Malta also experienced decreases (Supplementary Appendix 8; Figure 3d).

### 4. Temporal trends in regional DALYs burden

**Figure 4:**
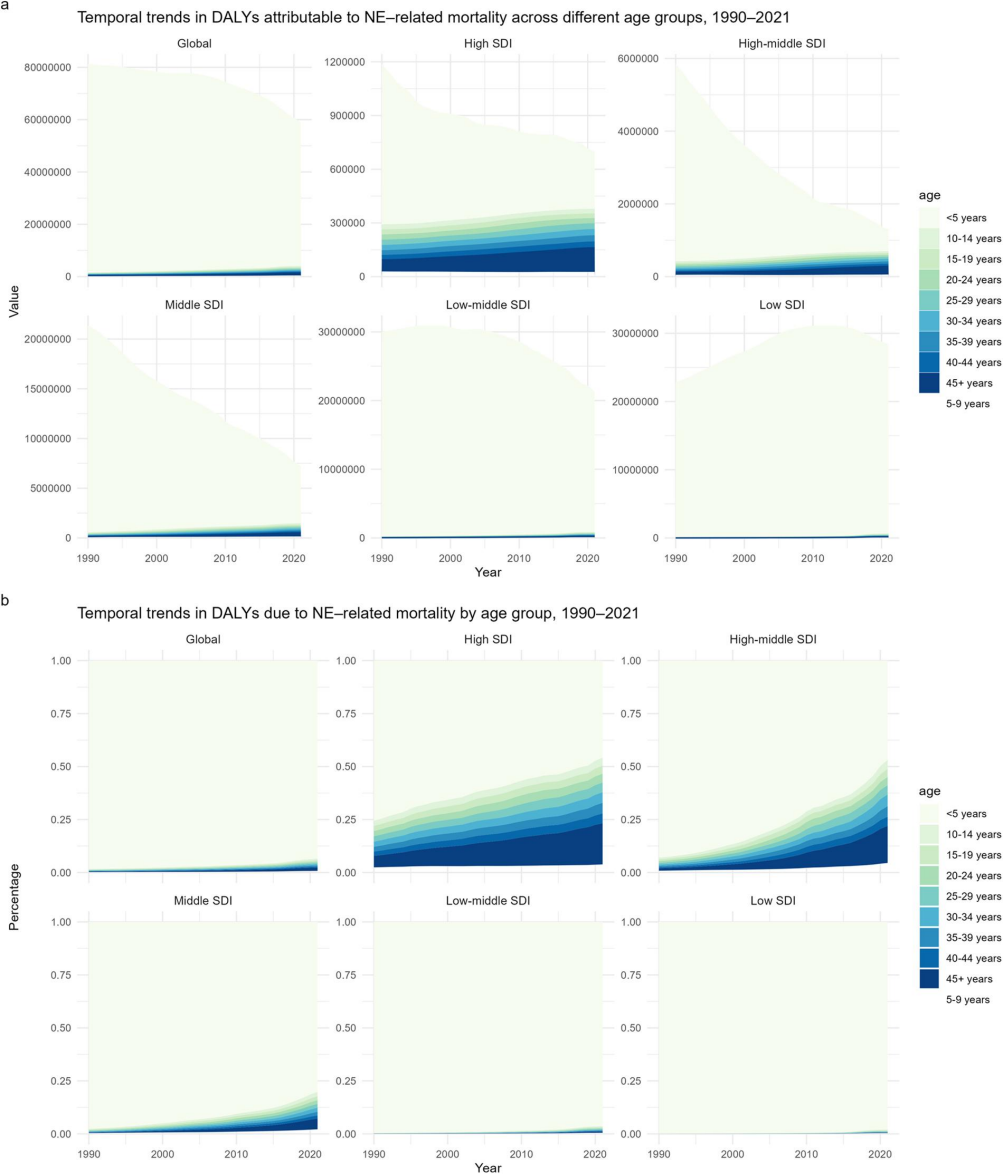
**(a)** Temporal change in the absolute cases of neonatal encephalopathy due to birth asphyxia and trauma DALYs across age groups, 1990–2021. **(b)** Temporal change in the relative proportion of Neonatal encephalopathy due to birth asphyxia and trauma across DALYs age groups, 1990–2021.

Globally, the disability-adjusted life years (DALYs) associated with NE declined substantially, from over 80 million in 1990 to below 60 million in 2021. This decline was especially pronounced in countries with high-middle and middle sociodemographic index (SDI) levels. In contrast, low-middle SDI countries began to show a downward trend after 1998, and a similar decline in low SDI countries became apparent after approximately 2010. Moreover, DALYs in individuals aged over 10 years exhibited an increasing trend worldwide, especially in high, high-middle, and middle SDI regions, with the rise most evident in populations aged 45 years and older. Conversely, low-middle and low SDI countries did not exhibit substantial changes in this age group. Children under the age of five contributed to the overwhelming majority of DALYs globally—exceeding 90%—but this proportion declined from 98.3% in 1990 to 93.5% in 2021. The declining proportion of DALYs in children under five was more prominent in countries with higher SDI levels. Specifically, in high SDI countries, the proportion declined from 75.6% to 45.8%, while high-middle SDI countries experienced the steepest drop—from 93% to 46.8%—and middle SDI countries saw a decrease from 97.6% to 80%. In summary, the higher the SDI level, the lower the proportion of DALYs attributed to children under 5 years of age (Figure 4).

**Figure 5:**
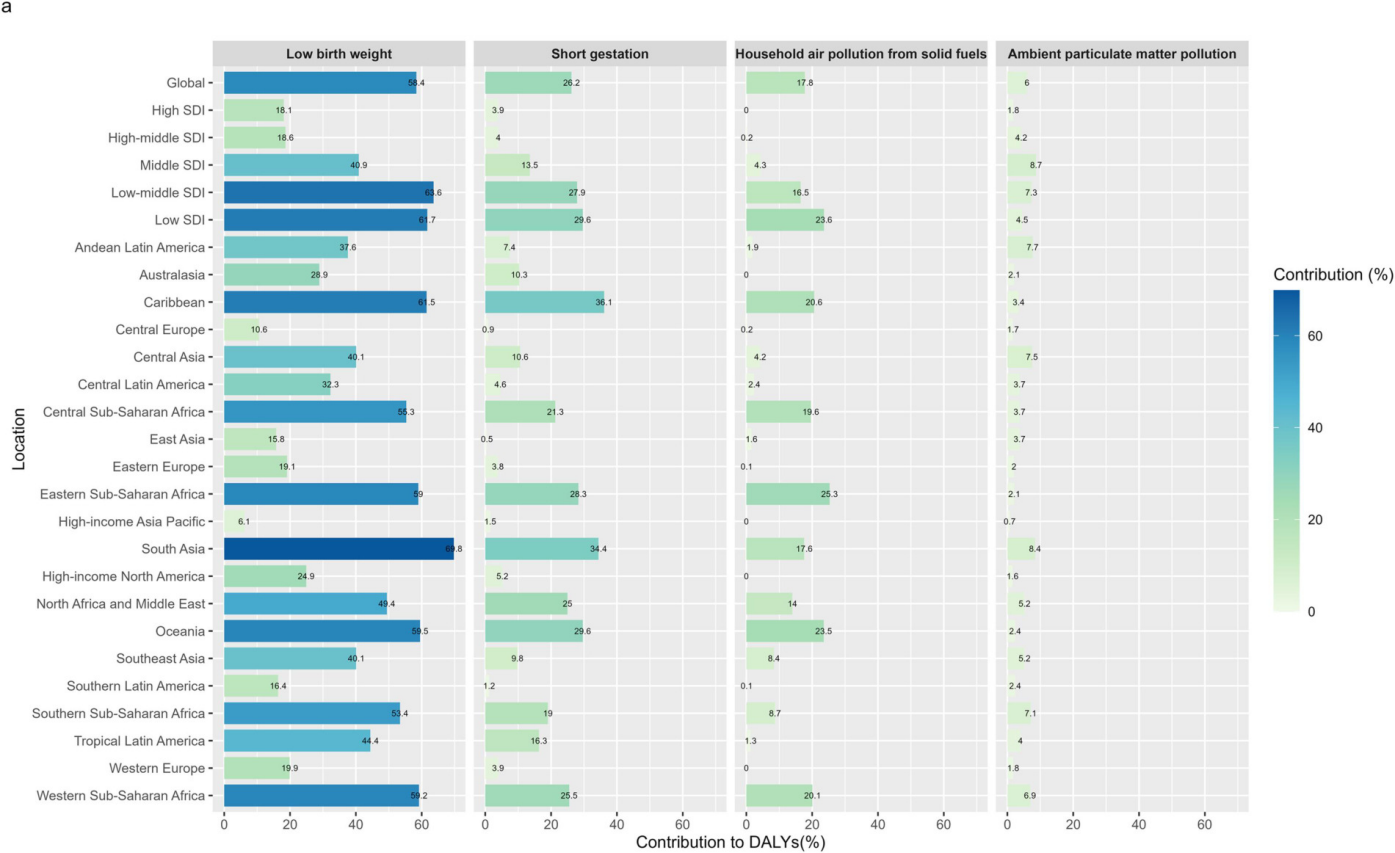
Risk factors of neonatal encephalopathy due to birth asphyxia and trauma DALYs across age groups, 2021.

### 5. Risk factors contribute to DALYs of NE

In 2021, low birth weight was the predominant global risk factor for disability-adjusted life years (DALYs) attributable to neonatal encephalopathy due to asphyxia and trauma, accounting for approximately 58.4%. Even in high and high-middle SDI countries, low birth weight remained a significant contributor, responsible for 18.1% and 18.6% of DALYs, respectively. Among the 21 global regions, South Asia (69.8%), the Caribbean (61.5%), and Western Sub-Saharan Africa (59.2%) were most heavily impacted by low birth weight. Preterm birth and indoor air pollution from solid fuel use were also important contributors, responsible for 26.2% and 17.8% of global DALYs, respectively. The regional patterns of these two risk factors closely mirrored those observed for low birth weight. In contrast, the contribution of ambient particulate matter pollution was comparatively modest, accounting for around 6% of global DALYs (Figure 5).

## Discussion

Neonatal encephalopathy (NE) is a clinically defined syndrome associated with an elevated risk of perinatal mortality and long-term neurological sequelae. Studies report that approximately 40% of affected neonates experience adverse outcomes (17). Hypoxic-ischemic encephalopathy is a principal cause of neonatal mortality and long-term disability, while trauma from mechanical forces applied to the head constitutes another major cause. Significant geographical disparities persist, resulting in a profoundly unequal global burden. This severe condition can result in mortality or sequelae including significant intellectual, cognitive, and motor impairments. The incidence of NE is substantially higher in resource-poor settings compared to developed nations. Consequently, a comprehensive analysis of NE burden attributable to asphyxia and trauma is of considerable clinical, epidemiological, and public health importance. Globally, asphyxia- and trauma-related NE has exhibited declining incidence, mortality, and DALY rates, but a rising prevalence. Although an acute condition, we report GBD prevalence—which captures both disease risk and survival—as a key metric. In combination, these data are crucial for public health planning, enabling local decision-makers to estimate annual caseloads and allocate medical resources to alleviate healthcare system strain.

Data from the Global Burden of Disease (GBD) study reveal that despite a global decrease in absolute NE case numbers due to asphyxia and trauma, the associated disease burden remains substantial. In 2021, there were approximately 1.06 million (95% UI:1,047,815–1,076,766)new NE cases globally, reflecting an 18.1% reduction from the 1.296 million (1,276,553–1,314,239) cases reported in 1990. These improvements are even more evident in age-standardized rates: the age-standardized incidence rate (ASIR) fell from 20.2(19.9–20.5) to 17.2(16.9–17.4)/100,000; the age-standardized mortality rate (ASMR) declined from 13.8 (12.7–15.7) to 9.8(8.3–11.7)/100,000; and the age-standardized disability-adjusted life year rate (ASDR) decreased from 1271 (1165–1444) to 932 (796–1102)/100,000, corresponding to an overall reduction of 26.7%.

The burden of NE follows a clear three-phase trajectory: a rapid decline between 1990 and 1998, a transient increase from 1999 to 2007, followed by a renewed downward trend from 2008 to 2021. These fluctuations likely reflect several factors: the expansion of primary healthcare systems in developing countries during the 1990s (18, 19), a period that saw a reduced baseline incidence; the collapse of healthcare infrastructures in conflict-affected areas during the late 1990s to early 2000s leading to increased burden; and renewed declines following enhanced maternal and child health investments under the UN Millennium Development Goals after 2005 (20, 21). Enhanced perinatal care has also contributed significantly to reducing NE-related mortality (22). Evidence indicates that the high incidence and mortality of NE are preventable through multidisciplinary team training and optimized obstetric management (23). Training to improve competency in interpreting electronic fetal monitoring can help prevent the occurrence of NE (24). Resuscitation and simulation-based team training have been shown to improve patient safety and mitigate neurodevelopmental sequelae (25). Additionally, marked sex differences exist in NE burden: in 2021, the incidence among males was 1.5 times that of females, and male mortality and DALYs were 1.4 times higher than those in females. The persistent higher NE burden in males aligns with the sex dimorphism observed post-NE in previous studies (26, 27). Meredith et al. reported that males are more susceptible to prenatal hypoxia, hemorrhage, and infections (28). Female animals show greater intrinsic neuroprotection and less NE-related neurological damage, with enhanced response to hypothermia. These sex-specific differences, involving hormonal and cellular mechanisms, may alter long-term neurological function and worsen the disease burden (29).

Applying the Sociodemographic Index (SDI) classification, NE mortality and DALYs have declined globally across all SDI strata, yet profound regional disparities persist. These inequalities are closely linked to SDI levels: low-SDI countries exhibit an ASDR fourteenfold higher than high-SDI countries, with a slower rate of DALYs reduction (EPAC: −0.73). Disparities are more pronounced in mortality rates, where the ASMR in low-SDI regions exceeds that of high-SDI regions by more than twenty-two times.

Sub-Saharan Africa (SSA) represents the global epicenter of NE burden, with 2021 ASMRs reported at 22.1 (18–26.4), 17.7(13.9–22.4), and 15.4 (12.3–19.2) per 100,000 population in West, Central, and East Africa, respectively. Additionally, South Asia's NE burden is considerable, with an ASMR of 12.9 (10.2–16.8)/100,000—over seven times higher than East Asia (1.7 [1.4–2.1]/100,000) and more than twentyfold that of Europe (0.8 [0.7–0.9] and 0.5 [0.4–0.6])/100,000 in Eastern and Central Europe, respectively). The ASDR data similarly reflect a severe burden. These statistics signify considerable mortality and chronic disability burdens; NE remains a major cause of neonatal death and severe cerebral palsy in low- and middle-income countries(LMICs) (30, 31). The Caribbean, Oceania, and Central Africa have seen the smallest declines in age-standardized mortality and DALY rates. Notably, the stagnation of progress in the Caribbean region may be attributed to the weak primary healthcare system, which fails to adequately cover maternal and neonatal populations (32). Additionally, this phenomenon is associated with regional social unrest, political instability, as well as inherent challenges in data collection and accessibility common in low Socio-Demographic Index (SDI) regions. While high-SDI regions show limited improvement due to already low baseline levels (33), Africa's low prevalence reflects high mortality from under-resourced health systems, with minimal survival among affected infants. Health system challenges—including weak infrastructure, scarce skilled birth attendants, and limited NICU capacity—contribute significantly to the NE burden (34). These challenges closely correlate with local environmental factors, lifestyle, primary healthcare coverage, and deficiencies in skilled birth attendance (35). Therapeutic hypothermia remains difficult to implement in LMICs, constrained by equipment shortages, training gaps, and the narrow 6-hour post-birth treatment window (36). Beyond hypothermia, comprehensive neurocritical care—including monitoring, seizure management, metabolic support, and nutrition—should be maintained for at least three days (37). Additionally, oxytocin use for labor in LMICs, while reducing labor duration and maternal mortality, increases risks of uterine hyperstimulation and fetal hypoxia, potentially contributing to NE (35).

Most countries show reduced NE incidence, DALYs, and mortality, reflecting global progress in perinatal care. In 2021, India, China, and Nigeria had the highest NE incidence, with distinct trajectories. China's incidence fell markedly due to neonatal special care nurseries since the 1990s, expanded health insurance by 2010, and standardized neurocritical care units by 2018, driving a steady decline from 1990 to 2021 (38–40). The improvement in china partially offset the grave NE situation in developing nations, underscoring limited progress elsewhere (41). India's high neonatal encephalopathy incidence and mortality are rooted in underlying structural determinants such as social inequality, malnutrition, and poor sanitation (42). Access to key therapeutic interventions like hypothermia remains limited and unevenly distributed. A 2017 survey indicated that only about half of neonatal units offered this treatment, with availability disproportionately concentrated in regions already demonstrating lower infant mortality rates (43). Furthermore, its effectiveness in local practice has been inconsistent, showing limited neuroprotective benefit and potential risks in some settings (44). Addressing this dual burden necessitates building upon existing health system frameworks while substantially increasing governmental investment in public health infrastructure (45). This challenge reflects a broader regional pattern across South Asia, where high neonatal mortality persists as seen in countries such as Pakistan, attributable to multiple risk factors, a widely dispersed rural population residing in remote areas, and an overall weak healthcare environment (46, 47). Nigeria faces similar challenges, with NE causing ∼40% of neonatal deaths, compounded by shortages of skilled providers and risks of sepsis and respiratory distress (48). Recent assessments in African nations reveal low performance in newborn care quality (49), Conflicts in South Sudan, Central African Republic, and Somalia have weakened health systems, leaving NE largely unmanaged, while fiscal cuts in Angola, Kenya, Ghana, India, and Mexico may worsen the NE burden (50–52). Presently, the International Federation of Gynecology and Obstetrics (FIGO) partners with stakeholders from heavily affected areas to establish the Fetal Growth Initiative, aiming to alleviate disease burden (53).

Globally, higher-income regions show lower or faster-declining NE incidence, mortality, and DALYs, attributed to stronger health investment (54). Notable variations exist: Japan reports lower NE mortality (moderate: 4.5%; severe: 32%) than Western nations, potentially due to societal norms favoring life support, leading to higher survival but increased prevalence of severe cases (55, 56). Taiwan shows atypical rises in mortality and DALYs. A systematic literature search was performed in PubMed and Web of Science databases using the key terms “Taiwan” and “neonatal encephalopathy (NE)”. Relevant studies from Taiwan primarily focused on NE-related topics, including perinatal stroke, cerebral palsy sequelae, and low birth weight. Therefore, we hypothesize that the abnormal elevation in NE incidence in Taiwan may be associated with optimization of data collection systems, potential Global Burden of Disease (GBD) data bias derived from previous studies, and variations in the application of disease diagnostic codes. Whether this observed increase reflects the true epidemiological status requires long-term prospective surveillance in future research (57). Globally, NE prevalence is increasing, especially in resource-limited countries like Ethiopia and Rwanda, reflecting both improved survival and persistent challenges (58, 59). In the GBD framework, prevalence estimates for acute neonatal conditions such as neonatal encephalopathy are derived from modeled incidence and short disease duration assumptions, rather than reflecting chronic disease states in the GBD framework. Consequently, increases in prevalence may occur alongside declining mortality, as improvements in neonatal survival allow more infants to be captured within the prevalence window. In low- and middle-income countries undergoing rapid health system strengthening (60, 61), such trends may also reflect enhanced case detection and refinements in GBD modeling inputs rather than a true increase in disease severity or duration. Additionally, the divergence between rising prevalence and falling mortality highlights a shift toward managing long-term functional outcomes and rehabilitation (21, 62, 63). DALYs are declining in under-fives but rising in older age groups, particularly in high-SDI countries, indicating historical gaps in perinatal care leading to chronic neurological sequelae (56, 62, 64). This increase in DALYs represents a lingering “legacy” of historically insufficient medical care. This reflects a fundamental shift in how NE is being addressed globally. Consequently, while the global demand for rehabilitation among NE survivors is steadily increasing, the availability of such resources remains highly unequal across regions (63–65). Further analysis indicated low birth weight as the dominant risk factor associated with DALYs (58%), with a disproportionate impact on low and lower-middle SDI countries. Preterm birth and indoor air pollution from solid fuel combustion were also strongly linked to the DALY burden of neonatal encephalopathy. While the GBD dataset provides valuable insights into numerous risk factors, it is crucial to recognize its inherent limitations in capturing the full spectrum of determinants associated with this condition. For example, critical elements such as socioeconomic deprivation, maternal undernutrition, quality of antenatal and intrapartum care, community-based childbirth practices, and infectious exposures are not systematically incorporated in the GBD framework—yet these factors are closely associated with neonatal encephalopathy risk. In certain contexts, high fertility rates, challenging living conditions for survivors, and shortages of skilled primary healthcare providers may further amplify the disease burden. Moreover, the lack of reliable air quality monitoring in low-SDI regions introduces uncertainty into pollution-related assessments. Although these factors exert relatively modest effects in high-SDI settings, their integration into prenatal care strategies and neonatal disease management remains essential for a comprehensive approach.

This study did not perform predictive modeling, as recent GBD projections align with stable historical NE trends. Based on GBD database, the current study has inherent limitations that should be recognized when interpreting the results The GBD data utilize refined statistical modeling (e.g., Bayesian hierarchical models) to improve estimate specificity (11), study showed 30 countries lacked comprehensive child mortality data during 2015–2021, and 41 countries had no available population census data between 2010 and 2021. The GBD framework necessitates extrapolation from surrogate indicators in similar countries, which may introduce regional bias (66). The 30-year span data in this study covers the transition period from ICD-9 to ICD-10 coding systems. Although no quantitative evidence of the coding transition's interference on neonatal encephalopathy (NE) statistics was obtained directly in this study, existing research has shown that the update of ICD coding systems significantly affects the epidemiological analysis of neurological diseases (67). Consequently, this coding transition is a potential confounding factor that should be considered when interpreting the temporal trends in disease burden estimates. A further limitation pertains to potential misclassification within the GBD modeling framework itself. “Encephalopathy due to birth asphyxia and trauma” combines neonatal hypoxic-ischemic encephalopathy and trauma-related encephalopathy. This aggregation, while methodologically consistent, merges conditions with distinct epidemiological profiles and introduces diagnostic uncertainty. Additionally, in low-SDI regions, risk factor data are often coarse, limiting granular exposure assessment. At last, low birth weight (LBW) is a non-independent risk factor: it frequently results from underlying conditions that may themselves influence NE risk, and its association with comorbidities complicates causal interpretation (68). Therefore, interpretations of LBW as a risk factor for NE should be made with caution. In summary, the aforementioned limitations may collectively constrain the precision and interpretability of the present study's findings. By addressing these aspects, subsequent investigations can enhance the validity of NE burden estimates and risk factor attribution, thereby providing more robust evidence to inform public health strategies for NE prevention and control worldwide.

## Conclusion

Between 1990 and 2021, the global incidence and mortality rates of asphyxia- and trauma-related neonatal encephalopathy (NE) have declined significantly. However, elevated incidence and mortality rates remain predominantly concentrated in low- and lower-middle Socio-Demographic Index (SDI) regions. In these areas, while improvements in prenatal care and nutritional support are essential, ensuring political stability and scaling up investments in healthcare infrastructure are equally critical. For middle- and high-SDI countries, there is an urgent need to expand and strategically allocate rehabilitation resources to address the substantial DALY (disability-adjusted life years) burden among NE survivors aged ≥10 years.

Tackling the long-term public health burden of NE necessitates global collaboration across health systems, knowledge exchange, and the implementation of context-specific interventions to reduce the overall burden and alleviate regional disparities. Building on the observed downward trends from 1990 to 2021, further sustained progress in reducing DALYs, incidence, and mortality can be expected through targeted global efforts aligned with regional contexts.

## Data Availability

The original contributions presented in the study are included in the article/Supplementary Material, further inquiries can be directed to the corresponding author.
