# Supplementary material for: Persistent inequities in neonatal encephalopathy: a 30-year global burden analysis (1990–2021)
Source: Front Pediatr. 2026 Jan 15;13:1694958. doi: 10.3389/fped.2025.1694958 (PMC12852322; doi:10.3389/fped.2025.1694958)
Supplement: Supplementary file 1 [file Table1.docx]

Appendix

Appendix1：Incidence, Death and DALYs Counts of Neonatal encephalopathy due to birth asphyxia and trauma

| measure | location | sex | age | cause | metric | year | val | upper | lower |
| --- | --- | --- | --- | --- | --- | --- | --- | --- | --- |
| Incidence | Global | Male | All ages | Neonatal encephalopathy due to birth asphyxia and trauma | Number | 1990 | 771836.3169029597 | 785417.6492258685 | 759824.1427766504 |
| Incidence | Global | Female | All ages | Neonatal encephalopathy due to birth asphyxia and trauma | Number | 1990 | 524054.7868918991 | 538318.1787117287 | 511319.8381646275 |
| Incidence | Global | Both | All ages | Neonatal encephalopathy due to birth asphyxia and trauma | Number | 1990 | 1295891.1037948576 | 1314238.816327619 | 1276552.8818827905 |
| Incidence | Global | Male | All ages | Neonatal encephalopathy due to birth asphyxia and trauma | Number | 2021 | 637774.0491396029 | 647596.347273072 | 629258.9129114215 |
| Incidence | Global | Female | All ages | Neonatal encephalopathy due to birth asphyxia and trauma | Number | 2021 | 423674.1030102508 | 433311.94090102153 | 414555.7705309442 |
| Incidence | Global | Both | All ages | Neonatal encephalopathy due to birth asphyxia and trauma | Number | 2021 | 1061448.1521498542 | 1076766.1119810052 | 1047815.174502404 |
| Deaths | Global | Male | All ages | Neonatal encephalopathy due to birth asphyxia and trauma | Number | 1990 | 517580.2062875213 | 594591.6915699301 | 462704.8621171644 |
| Deaths | Global | Female | All ages | Neonatal encephalopathy due to birth asphyxia and trauma | Number | 1990 | 365501.8496797368 | 422333.2739878869 | 324486.0771211541 |
| Deaths | Global | Both | All ages | Neonatal encephalopathy due to birth asphyxia and trauma | Number | 1990 | 883082.0559672584 | 1004097.1350131445 | 809080.1381785575 |
| Deaths | Global | Male | All ages | Neonatal encephalopathy due to birth asphyxia and trauma | Number | 2021 | 354306.17978908407 | 422742.42686548247 | 293053.94744341215 |
| Deaths | Global | Female | All ages | Neonatal encephalopathy due to birth asphyxia and trauma | Number | 2021 | 249299.44012844138 | 297288.91793779033 | 207717.77046551532 |
| Deaths | Global | Both | All ages | Neonatal encephalopathy due to birth asphyxia and trauma | Number | 2021 | 603605.6199175254 | 725270.8179207218 | 511190.6339044534 |
| DALYs (Disability-Adjusted Life Years) | Global | Male | All ages | Neonatal encephalopathy due to birth asphyxia and trauma | Number | 1990 | 47513074.84692518 | 54699631.96997666 | 42609788.83564628 |
| DALYs (Disability-Adjusted Life Years) | Global | Female | All ages | Neonatal encephalopathy due to birth asphyxia and trauma | Number | 1990 | 33507430.725790247 | 38645726.54403915 | 29736634.168319464 |
| DALYs (Disability-Adjusted Life Years) | Global | Both | All ages | Neonatal encephalopathy due to birth asphyxia and trauma | Number | 1990 | 81020505.57271534 | 92120587.36405286 | 74272388.03486219 |
| DALYs (Disability-Adjusted Life Years) | Global | Male | All ages | Neonatal encephalopathy due to birth asphyxia and trauma | Number | 2021 | 34414118.2514864 | 40569208.152256206 | 28910398.800973773 |
| DALYs (Disability-Adjusted Life Years) | Global | Female | All ages | Neonatal encephalopathy due to birth asphyxia and trauma | Number | 2021 | 24160972.331955213 | 28526985.73364923 | 20445156.735956345 |
| DALYs (Disability-Adjusted Life Years) | Global | Both | All ages | Neonatal encephalopathy due to birth asphyxia and trauma | Number | 2021 | 58575090.58344165 | 68835375.45214722 | 50032890.54147683 |

Appendix2：Incidence, Death and DALYs Rate of Neonatal encephalopathy due to birth asphyxia and trauma

| measure | location | sex | age | cause | metric | year | val | upper | lower |
| --- | --- | --- | --- | --- | --- | --- | --- | --- | --- |
| Deaths | Global | Female | All ages | Neonatal encephalopathy due to birth asphyxia and trauma | Rate | 1990 | 13.803563317003166 | 15.949862069035193 | 12.254559354357584 |
| Deaths | Global | Both | All ages | Neonatal encephalopathy due to birth asphyxia and trauma | Rate | 1990 | 16.55688798204323 | 18.82579730294005 | 15.169427490682821 |
| Deaths | Global | Male | All ages | Neonatal encephalopathy due to birth asphyxia and trauma | Rate | 2021 | 8.948500930845594 | 10.676954611871677 | 7.401489646741252 |
| Deaths | Global | Female | All ages | Neonatal encephalopathy due to birth asphyxia and trauma | Rate | 2021 | 6.340332101500782 | 7.560829133231427 | 5.282802269656711 |
| Deaths | Global | Both | All ages | Neonatal encephalopathy due to birth asphyxia and trauma | Rate | 2021 | 7.648949386932102 | 9.190702662530642 | 6.477857655373002 |
| DALYs (Disability-Adjusted Life Years) | Global | Male | All ages | Neonatal encephalopathy due to birth asphyxia and trauma | Rate | 1990 | 1769.0849296970496 | 2036.6666415470472 | 1586.5177223231985 |
| DALYs (Disability-Adjusted Life Years) | Global | Female | All ages | Neonatal encephalopathy due to birth asphyxia and trauma | Rate | 1990 | 1265.4435046466053 | 1459.4966721773278 | 1123.035390755543 |
| DALYs (Disability-Adjusted Life Years) | Global | Both | All ages | Neonatal encephalopathy due to birth asphyxia and trauma | Rate | 1990 | 1519.051854752777 | 1727.1670684736457 | 1392.5315326502964 |
| DALYs (Disability-Adjusted Life Years) | Global | Male | All ages | Neonatal encephalopathy due to birth asphyxia and trauma | Rate | 2021 | 869.1769626795083 | 1024.632415754771 | 730.1727865306676 |
| DALYs (Disability-Adjusted Life Years) | Global | Female | All ages | Neonatal encephalopathy due to birth asphyxia and trauma | Rate | 2021 | 614.4762635681967 | 725.515321305675 | 519.973424354314 |
| DALYs (Disability-Adjusted Life Years) | Global | Both | All ages | Neonatal encephalopathy due to birth asphyxia and trauma | Rate | 2021 | 742.2692705692948 | 872.2886028395247 | 634.0216770777606 |
| Incidence | Global | Male | All ages | Neonatal encephalopathy due to birth asphyxia and trauma | Rate | 1990 | 28.7382789 | 29.2439614 | 28.291021882640003 |
| Incidence | Global | Female | All ages | Neonatal encephalopathy due to birth asphyxia and trauma | Rate | 1990 | 19.79148242 | 20.330154474049998 | 19.31053363 |
| Incidence | Global | Both | All ages | Neonatal encephalopathy due to birth asphyxia and trauma | Rate | 1990 | 24.29663665 | 24.640637544999997 | 23.934064706290002 |
| Incidence | Global | Male | All ages | Neonatal encephalopathy due to birth asphyxia and trauma | Rate | 2021 | 16.10788069 | 16.35595665 | 15.89281895 |
| Incidence | Global | Female | All ages | Neonatal encephalopathy due to birth asphyxia and trauma | Rate | 2021 | 10.77513257 | 11.02024781 | 10.54322969 |
| Incidence | Global | Both | All ages | Neonatal encephalopathy due to birth asphyxia and trauma | Rate | 2021 | 13.45077469 | 13.644885369410002 | 13.278016261210002 |

Appendix3：ASIR, ASDR andASMR of Neonatal encephalopathy due to birth asphyxia and trauma

| measure | location | sex | age | cause | metric | year | val | upper | lower |
| --- | --- | --- | --- | --- | --- | --- | --- | --- | --- |
| Deaths | Global | Male | Age-standardized | Neonatal encephalopathy due to birth asphyxia and trauma | Rate | 1990 | 15.626904401577834 | 17.957174092337524 | 13.973784993741793 |
| Deaths | Global | Female | Age-standardized | Neonatal encephalopathy due to birth asphyxia and trauma | Rate | 1990 | 11.85639789566367 | 13.702702247881195 | 10.523963848506183 |
| Deaths | Global | Both | Age-standardized | Neonatal encephalopathy due to birth asphyxia and trauma | Rate | 1990 | 13.809339171560358 | 15.705093312235366 | 12.651790181181108 |
| Deaths | Global | Male | Age-standardized | Neonatal encephalopathy due to birth asphyxia and trauma | Rate | 2021 | 11.067617537380679 | 13.205892108704171 | 9.154663698572655 |
| Deaths | Global | Female | Age-standardized | Neonatal encephalopathy due to birth asphyxia and trauma | Rate | 2021 | 8.338966498039602 | 9.94430379369634 | 6.947507339062299 |
| Deaths | Global | Both | Age-standardized | Neonatal encephalopathy due to birth asphyxia and trauma | Rate | 2021 | 9.750062833830961 | 11.714638896504367 | 8.257515474447692 |
| DALYs (Disability-Adjusted Life Years) | Global | Male | Age-standardized | Neonatal encephalopathy due to birth asphyxia and trauma | Rate | 1990 | 1439.3176723345998 | 1657.6141916781917 | 1291.0110910839958 |
| DALYs (Disability-Adjusted Life Years) | Global | Female | Age-standardized | Neonatal encephalopathy due to birth asphyxia and trauma | Rate | 1990 | 1089.6430969796731 | 1256.7836337930007 | 967.9311549044778 |
| DALYs (Disability-Adjusted Life Years) | Global | Both | Age-standardized | Neonatal encephalopathy due to birth asphyxia and trauma | Rate | 1990 | 1270.670100828023 | 1443.7135401633147 | 1164.5938645030512 |
| DALYs (Disability-Adjusted Life Years) | Global | Male | Age-standardized | Neonatal encephalopathy due to birth asphyxia and trauma | Rate | 2021 | 1060.173814559686 | 1252.5846666310683 | 889.766181737332 |
| DALYs (Disability-Adjusted Life Years) | Global | Female | Age-standardized | Neonatal encephalopathy due to birth asphyxia and trauma | Rate | 2021 | 795.3550083859564 | 942.2738207391361 | 670.5129897820977 |
| DALYs (Disability-Adjusted Life Years) | Global | Both | Age-standardized | Neonatal encephalopathy due to birth asphyxia and trauma | Rate | 2021 | 932.1426617145408 | 1101.5439417330085 | 796.2941174480817 |
| Incidence | Global | Male | Age-standardized | Neonatal encephalopathy due to birth asphyxia and trauma | Rate | 1990 | 23.24838976 | 23.65747146 | 22.8865725 |
| Incidence | Global | Female | Age-standardized | Neonatal encephalopathy due to birth asphyxia and trauma | Rate | 1990 | 16.97061884 | 17.43251441 | 16.55821928 |
| Incidence | Global | Both | Age-standardized | Neonatal encephalopathy due to birth asphyxia and trauma | Rate | 1990 | 20.22311987 | 20.50944639 | 19.92133589 |
| Incidence | Global | Male | Age-standardized | Neonatal encephalopathy due to birth asphyxia and trauma | Rate | 2021 | 19.93396401 | 20.240965118149997 | 19.66781894 |
| Incidence | Global | Female | Age-standardized | Neonatal encephalopathy due to birth asphyxia and trauma | Rate | 2021 | 14.18448624 | 14.50715826 | 13.87920711 |
| Incidence | Global | Both | Age-standardized | Neonatal encephalopathy due to birth asphyxia and trauma | Rate | 2021 | 17.15799585 | 17.40560615 | 16.937622793270002 |

Appendix4

| measure | location | sex | age | cause | metric | year | val | upper | lower |
| --- | --- | --- | --- | --- | --- | --- | --- | --- | --- |
| Incidence | Republic of India | Both | All ages | Neonatal encephalopathy due to birth asphyxia and trauma | Number | 2021 | 169064.4 | 178943.4 | 159957.6 |
| Incidence | Federal Republic of Nigeria | Both | All ages | Neonatal encephalopathy due to birth asphyxia and trauma | Number | 2021 | 90497.7 | 96088.37 | 85073.54 |
| Incidence | People's Republic of China | Both | All ages | Neonatal encephalopathy due to birth asphyxia and trauma | Number | 2021 | 74198.78 | 78261.43 | 70060.55 |
| Prevalence | Kingdom of Thailand | Both | Age-standardized | Neonatal encephalopathy due to birth asphyxia and trauma | Rate | 2021 | 517.00 | 590.17 | 452.87 |
| Prevalence | United Mexican States | Both | Age-standardized | Neonatal encephalopathy due to birth asphyxia and trauma | Rate | 2021 | 447.52 | 507.06 | 389.01 |
| Prevalence | North Macedonia | Both | Age-standardized | Neonatal encephalopathy due to birth asphyxia and trauma | Rate | 2021 | 422.94 | 480.42 | 369.11 |
| Prevalence | Central African Republic | Both | Age-standardized | Neonatal encephalopathy due to birth asphyxia and trauma | Rate | 2021 | 75.43 | 98.691 | 56.55 |
| Prevalence | Portuguese Republic | Both | Age-standardized | Neonatal encephalopathy due to birth asphyxia and trauma | Rate | 2021 | 82.22 | 93.26 | 72.99 |
| Prevalence | Islamic Republic of Afghanistan | Both | Age-standardized | Neonatal encephalopathy due to birth asphyxia and trauma | Rate | 2021 | 82.89 | 101.24 | 66.53 |
| Deaths | Federal Republic of Nigeria | Both | All ages | Neonatal encephalopathy due to birth asphyxia and trauma | Number | 2021 | 100304.6 | 123896.3 | 78778.05 |
| Deaths | Islamic Republic of Pakistan | Both | All ages | Neonatal encephalopathy due to birth asphyxia and trauma | Number | 2021 | 96081.87 | 119862.7 | 73472.28 |
| Deaths | Republic of India | Both | All ages | Neonatal encephalopathy due to birth asphyxia and trauma | Number | 2021 | 77652.04 | 126852.3 | 53706.24 |
| Incidence | Federal Republic of Somalia | Both | Age-standardized | Neonatal encephalopathy due to birth asphyxia and trauma | Rate | 2021 | 56.13184 | 59.43965 | 52.71173 |
| Incidence | Republic of Burundi | Both | Age-standardized | Neonatal encephalopathy due to birth asphyxia and trauma | Rate | 2021 | 39.56614 | 41.83759 | 37.21664 |
| Incidence | Republic of Uganda | Both | Age-standardized | Neonatal encephalopathy due to birth asphyxia and trauma | Rate | 2021 | 38.33545 | 40.51086 | 36.22578 |
| Incidence | Portuguese Republic | Both | Age-standardized | Neonatal encephalopathy due to birth asphyxia and trauma | Rate | 2021 | 3.222575 | 3.404366 | 3.043992 |
| Incidence | Kingdom of Belgium | Both | Age-standardized | Neonatal encephalopathy due to birth asphyxia and trauma | Rate | 2021 | 3.372002 | 3.556342 | 3.191446 |
| Incidence | Australia | Both | Age-standardized | Neonatal encephalopathy due to birth asphyxia and trauma | Rate | 2021 | 3.932626 | 4.180999 | 3.706047 |
| Deaths | Islamic Republic of Pakistan | Both | Age-standardized | Neonatal encephalopathy due to birth asphyxia and trauma | Rate | 2021 | 32.21286 | 40.18587 | 24.63573 |
| Deaths | Central African Republic | Both | Age-standardized | Neonatal encephalopathy due to birth asphyxia and trauma | Rate | 2021 | 29.53508 | 38.79704 | 21.6884 |
| Deaths | Republic of South Sudan | Both | Age-standardized | Neonatal encephalopathy due to birth asphyxia and trauma | Rate | 2021 | 28.85425 | 40.47415 | 19.18472 |
| Deaths | Republic of Slovenia | Both | Age-standardized | Neonatal encephalopathy due to birth asphyxia and trauma | Rate | 2021 | 0.12695 | 0.150916 | 0.106242 |
| Deaths | Principality of Andorra | Both | Age-standardized | Neonatal encephalopathy due to birth asphyxia and trauma | Rate | 2021 | 0.135775 | 0.210909 | 0.067847 |
| Deaths | United Arab Emirates | Both | Age-standardized | Neonatal encephalopathy due to birth asphyxia and trauma | Rate | 2021 | 0.177566 | 0.249804 | 0.120039 |
| DALYs (Disability-Adjusted Life Years) | Islamic Republic of Pakistan | Both | Age-standardized | Neonatal encephalopathy due to birth asphyxia and trauma | Rate | 2021 | 2927.957 | 3644.698 | 2245.01 |
| DALYs (Disability-Adjusted Life Years) | Central African Republic | Both | Age-standardized | Neonatal encephalopathy due to birth asphyxia and trauma | Rate | 2021 | 2686.955 | 3528.468 | 1995.23 |
| DALYs (Disability-Adjusted Life Years) | Republic of South Sudan | Both | Age-standardized | Neonatal encephalopathy due to birth asphyxia and trauma | Rate | 2021 | 2660.325 | 3713.313 | 1788.777 |
| DALYs (Disability-Adjusted Life Years) | Principality of Andorra | Both | Age-standardized | Neonatal encephalopathy due to birth asphyxia and trauma | Rate | 2021 | 43.75604 | 56.64984 | 32.24935 |
| DALYs (Disability-Adjusted Life Years) | Republic of Poland | Both | Age-standardized | Neonatal encephalopathy due to birth asphyxia and trauma | Rate | 2021 | 46.0736 | 55.20465 | 37.31077 |
| DALYs (Disability-Adjusted Life Years) | State of Qatar | Both | Age-standardized | Neonatal encephalopathy due to birth asphyxia and trauma | Rate | 2021 | 46.88225 | 59.67608 | 35.73477 |

Appendix5

| measure | | location | | | cause | | val | | | lower | | | upper | |
| --- | --- | --- | --- | --- | --- | --- | --- | --- | --- | --- | --- | --- | --- | --- |
| EAPC for incidence | | Kuwait | | | Neonatal encephalopathy due to birth asphyxia and trauma | | 1.25 | | | 0.77 | | | 1.73 | |
| EPAC for incidence | | American Samoa | | | Neonatal encephalopathy due to birth asphyxia and trauma | | 1.19 | | | 0.56 | | | 1.82 | |
| EPAC for incidence | | Northern Mariana Islands | | | Neonatal encephalopathy due to birth asphyxia and trauma | | 1.12 | | | 0.45 | | | 1.8 | |
| EPAC for incidence | | Timor-Leste | | | Neonatal encephalopathy due to birth asphyxia and trauma | | -2.77 | | | -3.08 | | | -2.46 | |
| EPAC for incidence | | Equatorial Guinea | | | Neonatal encephalopathy due to birth asphyxia and trauma | | -2.57 | | | -2.98 | | | -2.17 | |
| EPAC for incidence | | Cambodia | | | Neonatal encephalopathy due to birth asphyxia and trauma | | -2.49 | | | -2.71 | | | -2.26 | |
| measure | location | | sex | age | | cause | | metric | year | | val | upper | | lower |
| Incidence | Democratic Republic of Timor-Leste | | Both | Age-standardized | | Neonatal encephalopathy due to birth asphyxia and trauma | | Rate | 1990 | | 41.47094 | 43.99319 | | 39.23105 |
| Incidence | Democratic Republic of Timor-Leste | | Both | Age-standardized | | Neonatal encephalopathy due to birth asphyxia and trauma | | Rate | 2021 | | 20.18219 | 21.33935 | | 19.0197 |
| Incidence | Republic of Equatorial Guinea | | Both | Age-standardized | | Neonatal encephalopathy due to birth asphyxia and trauma | | Rate | 1990 | | 34.25715 | 36.2837 | | 32.38461 |
| Incidence | Republic of Equatorial Guinea | | Both | Age-standardized | | Neonatal encephalopathy due to birth asphyxia and trauma | | Rate | 2021 | | 17.89633 | 18.97769 | | 16.88064 |
| Incidence | Kingdom of Cambodia | | Both | Age-standardized | | Neonatal encephalopathy due to birth asphyxia and trauma | | Rate | 1990 | | 38.59848 | 40.96464 | | 36.37703 |
| Incidence | Kingdom of Cambodia | | Both | Age-standardized | | Neonatal encephalopathy due to birth asphyxia and trauma | | Rate | 2021 | | 19.36271 | 20.56787 | | 18.34733 |
| Incidence | State of Kuwait | | Both | Age-standardized | | Neonatal encephalopathy due to birth asphyxia and trauma | | Rate | 2021 | | 14.7133 | 15.5311 | | 13.91627 |
| Incidence | State of Kuwait | | Both | Age-standardized | | Neonatal encephalopathy due to birth asphyxia and trauma | | Rate | 1990 | | 10.71459 | 11.35034 | | 10.11368 |
| Incidence | American Samoa | | Both | Age-standardized | | Neonatal encephalopathy due to birth asphyxia and trauma | | Rate | 2021 | | 8.931311 | 9.480085 | | 8.497245 |
| Incidence | American Samoa | | Both | Age-standardized | | Neonatal encephalopathy due to birth asphyxia and trauma | | Rate | 1990 | | 6.768738 | 7.156127 | | 6.398637 |
| Incidence | Northern Mariana Islands | | Both | Age-standardized | | Neonatal encephalopathy due to birth asphyxia and trauma | | Rate | 2021 | | 7.672885 | 8.095455 | | 7.288051 |
| Incidence | Northern Mariana Islands | | Both | Age-standardized | | Neonatal encephalopathy due to birth asphyxia and trauma | | Rate | 1990 | | 6.011004 | 6.36865 | | 5.6982 |

Appendix6

| measure | | location | | | cause | | val | | | lower | | | upper | |
| --- | --- | --- | --- | --- | --- | --- | --- | --- | --- | --- | --- | --- | --- | --- |
| EAPC for Death | | Taiwan (Province of China) | | | Neonatal encephalopathy due to birth asphyxia and trauma | | 3.3 | | | 2.77 | | | 3.82 | |
| EAPC for Death | | Dominica | | | Neonatal encephalopathy due to birth asphyxia and trauma | | 1.66 | | | 1.24 | | | 2.08 | |
| EAPC for Death | | South Sudan | | | Neonatal encephalopathy due to birth asphyxia and trauma | | 1.03 | | | 0.68 | | | 1.38 | |
| EAPC for Death | | Georgia | | | Neonatal encephalopathy due to birth asphyxia and trauma | | -10.34 | | | -11.15 | | | -9.53 | |
| EAPC for Death | | Estonia | | | Neonatal encephalopathy due to birth asphyxia and trauma | | -10.31 | | | -10.51 | | | -10.1 | |
| EAPC for Death | | Saudi Arabia | | | Neonatal encephalopathy due to birth asphyxia and trauma | | -8.61 | | | -8.87 | | | -8.34 | |
| measure | location | | sex | age | | cause | | metric | year | | val | upper | | lower |
| Deaths | Georgia | | Both | Age-standardized | | Neonatal encephalopathy due to birth asphyxia and trauma | | Rate | 1990 | | 21.29973 | 24.89032 | | 18.01606 |
| Deaths | Georgia | | Both | Age-standardized | | Neonatal encephalopathy due to birth asphyxia and trauma | | Rate | 2021 | | 1.267648 | 1.631337 | | 0.990197 |
| Deaths | Republic of Estonia | | Both | Age-standardized | | Neonatal encephalopathy due to birth asphyxia and trauma | | Rate | 1990 | | 4.132625 | 4.58238 | | 3.748952 |
| Deaths | Republic of Estonia | | Both | Age-standardized | | Neonatal encephalopathy due to birth asphyxia and trauma | | Rate | 2021 | | 0.182427 | 0.211171 | | 0.156563 |
| Deaths | Kingdom of Saudi Arabia | | Both | Age-standardized | | Neonatal encephalopathy due to birth asphyxia and trauma | | Rate | 1990 | | 5.911284 | 7.983929 | | 4.222124 |
| Deaths | Kingdom of Saudi Arabia | | Both | Age-standardized | | Neonatal encephalopathy due to birth asphyxia and trauma | | Rate | 2021 | | 0.406495 | 0.568031 | | 0.272191 |
| Deaths | Commonwealth of Dominica | | Both | Age-standardized | | Neonatal encephalopathy due to birth asphyxia and trauma | | Rate | 1990 | | 7.195173 | 9.867875 | | 5.294922 |
| Deaths | Commonwealth of Dominica | | Both | Age-standardized | | Neonatal encephalopathy due to birth asphyxia and trauma | | Rate | 2021 | | 9.850252 | 14.187 | | 6.270016 |
| Deaths | Republic of South Sudan | | Both | Age-standardized | | Neonatal encephalopathy due to birth asphyxia and trauma | | Rate | 2021 | | 28.85425 | 40.47415 | | 19.18472 |
| Deaths | Republic of South Sudan | | Both | Age-standardized | | Neonatal encephalopathy due to birth asphyxia and trauma | | Rate | 1990 | | 21.8843 | 29.55923 | | 14.5799 |
| Deaths | Taiwan (Province of China) | | Both | Age-standardized | | Neonatal encephalopathy due to birth asphyxia and trauma | | Rate | 1990 | | 0.222017 | 0.246317 | | 0.198991 |
| Deaths | Taiwan (Province of China) | | Both | Age-standardized | | Neonatal encephalopathy due to birth asphyxia and trauma | | Rate | 2021 | | 0.368727 | 0.431853 | | 0.301444 |

Appendix7

| measure | | location | | | cause | | val | | | lower | | | upper | |
| --- | --- | --- | --- | --- | --- | --- | --- | --- | --- | --- | --- | --- | --- | --- |
| EAPC for DALYs | | Taiwan (Province of China) | | | Neonatal encephalopathy due to birth asphyxia and trauma | | 1.55 | | | 1.24 | | | 1.86 | |
| EAPC for DALYs | | Dominica | | | Neonatal encephalopathy due to birth asphyxia and trauma | | 1.5 | | | 1.06 | | | 1.94 | |
| EAPC for DALYs | | South Sudan | | | Neonatal encephalopathy due to birth asphyxia and trauma | | 1.08 | | | 0.73 | | | 1.43 | |
| EAPC for DALYs | | Georgia | | | Neonatal encephalopathy due to birth asphyxia and trauma | | -9.08 | | | -9.82 | | | -8.34 | |
| EAPC for DALYs | | Estonia | | | Neonatal encephalopathy due to birth asphyxia and trauma | | -6.72 | | | -7.01 | | | -6.42 | |
| EAPC for DALYs | | Poland | | | Neonatal encephalopathy due to birth asphyxia and trauma | | -6.37 | | | -6.59 | | | -6.14 | |
| measure | location | | sex | age | | cause | | metric | year | | val | upper | | lower |
| DALYs | Georgia | | Both | Age-standardized | | Neonatal encephalopathy due to birth asphyxia and trauma | | Rate | 1990 | | 1954.732 | 2277.549 | | 1663.999 |
| DALYs | Georgia | | Both | Age-standardized | | Neonatal encephalopathy due to birth asphyxia and trauma | | Rate | 2021 | | 184.3761 | 221.8208 | | 152.4206 |
| DALYs | Republic of Estonia | | Both | Age-standardized | | Neonatal encephalopathy due to birth asphyxia and trauma | | Rate | 2021 | | 60.70817 | 75.99994 | | 48.00416 |
| DALYs | Republic of Estonia | | Both | Age-standardized | | Neonatal encephalopathy due to birth asphyxia and trauma | | Rate | 1990 | | 405.9382 | 448.1572 | | 367.9182 |
| DALYs | Republic of Poland | | Both | Age-standardized | | Neonatal encephalopathy due to birth asphyxia and trauma | | Rate | 2021 | | 46.0736 | 55.20465 | | 37.31077 |
| DALYs | Republic of Poland | | Both | Age-standardized | | Neonatal encephalopathy due to birth asphyxia and trauma | | Rate | 1990 | | 280.0079 | 311.8599 | | 253.161 |
| DALYs | Taiwan (Province of China) | | Both | Age-standardized | | Neonatal encephalopathy due to birth asphyxia and trauma | | Rate | 2021 | | 81.96054 | 99.9173 | | 66.97309 |
| DALYs | Taiwan (Province of China) | | Both | Age-standardized | | Neonatal encephalopathy due to birth asphyxia and trauma | | Rate | 1990 | | 63.96504 | 85.14782 | | 48.19449 |
| DALYs | Commonwealth of Dominica | | Both | Age-standardized | | Neonatal encephalopathy due to birth asphyxia and trauma | | Rate | 1990 | | 717.895 | 963.3477 | | 540.9921 |
| DALYs | Commonwealth of Dominica | | Both | Age-standardized | | Neonatal encephalopathy due to birth asphyxia and trauma | | Rate | 2021 | | 954.8902 | 1346.074 | | 633.3827 |
| DALYs | Republic of South Sudan | | Both | Age-standardized | | Neonatal encephalopathy due to birth asphyxia and trauma | | Rate | 1990 | | 1974.368 | 2663.272 | | 1317.824 |
| DALYs | Republic of South Sudan | | Both | Age-standardized | | Neonatal encephalopathy due to birth asphyxia and trauma | | Rate | 2021 | | 2660.325 | 3713.313 | | 1788.777 |

Appendix8

| measure | | location | | | cause | | val | | | lower | | | upper | |
| --- | --- | --- | --- | --- | --- | --- | --- | --- | --- | --- | --- | --- | --- | --- |
| EAPC for Prevalence | | Australia | | | Neonatal encephalopathy due to birth asphyxia and trauma | | -1.06 | | | -1.5 | | | -0.61 | |
| EAPC for Prevalence | | Portugal | | | Neonatal encephalopathy due to birth asphyxia and trauma | | -0.75 | | | -1.31 | | | -0.18 | |
| EAPC for Prevalence | | Malta | | | Neonatal encephalopathy due to birth asphyxia and trauma | | -0.49 | | | -1.01 | | | 0.02 | |
| EAPC for Prevalence | | Ethiopia | | | Neonatal encephalopathy due to birth asphyxia and trauma | | 12.23 | | | 11.97 | | | 12.48 | |
| EAPC for Prevalence | | Rwanda | | | Neonatal encephalopathy due to birth asphyxia and trauma | | 10.39 | | | 10.04 | | | 10.74 | |
| EAPC for Prevalence | | Equatorial Guinea | | | Neonatal encephalopathy due to birth asphyxia and trauma | | 9.77 | | | 9.23 | | | 10.32 | |
| measure | location | | sex | age | | cause | | metric | year | | val | upper | | lower |
| Prevalence | Federal Democratic Republic of Ethiopia | | Both | Age-standardized | | Neonatal encephalopathy due to birth asphyxia and trauma | | Rate | 1990 | | 11.95006 | 24.30023 | | 4.774148 |
| Prevalence | Federal Democratic Republic of Ethiopia | | Both | Age-standardized | | Neonatal encephalopathy due to birth asphyxia and trauma | | Rate | 2021 | | 325.4031 | 406.1984 | | 256.8007 |
| Prevalence | Republic of Rwanda | | Both | Age-standardized | | Neonatal encephalopathy due to birth asphyxia and trauma | | Rate | 1990 | | 23.96047 | 50.73797 | | 9.393834 |
| Prevalence | Republic of Rwanda | | Both | Age-standardized | | Neonatal encephalopathy due to birth asphyxia and trauma | | Rate | 2021 | | 303.2338 | 366.8135 | | 246.6822 |
| Prevalence | Republic of Equatorial Guinea | | Both | Age-standardized | | Neonatal encephalopathy due to birth asphyxia and trauma | | Rate | 2021 | | 252.2781 | 292.5208 | | 214.3334 |
| Prevalence | Republic of Equatorial Guinea | | Both | Age-standardized | | Neonatal encephalopathy due to birth asphyxia and trauma | | Rate | 1990 | | 20.41561 | 44.91143 | | 8.131348 |
| Prevalence | Australia | | Both | Age-standardized | | Neonatal encephalopathy due to birth asphyxia and trauma | | Rate | 2021 | | 99.94436 | 114.3064 | | 87.99937 |
| Prevalence | Australia | | Both | Age-standardized | | Neonatal encephalopathy due to birth asphyxia and trauma | | Rate | 1990 | | 128.6956 | 151.3377 | | 108.3134 |
| Prevalence | Portuguese Republic | | Both | Age-standardized | | Neonatal encephalopathy due to birth asphyxia and trauma | | Rate | 2021 | | 82.21724 | 93.2602 | | 72.99094 |
| Prevalence | Portuguese Republic | | Both | Age-standardized | | Neonatal encephalopathy due to birth asphyxia and trauma | | Rate | 1990 | | 96.42634 | 115.4544 | | 78.68668 |
| Prevalence | Republic of Malta | | Both | Age-standardized | | Neonatal encephalopathy due to birth asphyxia and trauma | | Rate | 2021 | | 167.1182 | 189.4938 | | 148.2603 |
| Prevalence | Republic of Malta | | Both | Age-standardized | | Neonatal encephalopathy due to birth asphyxia and trauma | | Rate | 1990 | | 194.9707 | 229.0593 | | 165.3997 |
